# Quantifying excess deaths related to heatwaves under climate change scenarios: A multicountry time series modelling study

**DOI:** 10.1371/journal.pmed.1002629

**Published:** 2018-07-31

**Authors:** Yuming Guo, Antonio Gasparrini, Shanshan Li, Francesco Sera, Ana Maria Vicedo-Cabrera, Micheline de Sousa Zanotti Stagliorio Coelho, Paulo Hilario Nascimento Saldiva, Eric Lavigne, Benjawan Tawatsupa, Kornwipa Punnasiri, Ala Overcenco, Patricia Matus Correa, Nicolas Valdes Ortega, Haidong Kan, Samuel Osorio, Jouni J. K. Jaakkola, Niilo R. I. Ryti, Patrick G. Goodman, Ariana Zeka, Paola Michelozzi, Matteo Scortichini, Masahiro Hashizume, Yasushi Honda, Xerxes Seposo, Ho Kim, Aurelio Tobias, Carmen Íñiguez, Bertil Forsberg, Daniel Oudin Åström, Yue Leon Guo, Bing-Yu Chen, Antonella Zanobetti, Joel Schwartz, Tran Ngoc Dang, Dung Do Van, Michelle L. Bell, Ben Armstrong, Kristie L. Ebi, Shilu Tong

**Affiliations:** 1 Department of Epidemiology and Preventive Medicine, School of Public Health and Preventive Medicine, Monash University, Melbourne, Australia; 2 Department of Public Health, Environments and Society, London School of Hygiene & Tropical Medicine, London, United Kingdom; 3 Institute of Advanced Studies, University of São Paulo, São Paulo, Brazil; 4 Faculty of Sciences, University of Technology Sydney, Sydney, Australia; 5 School of Epidemiology & Public Health, University of Ottawa, Ottawa, Canada; 6 Health Impact Assessment Division, Department of Health, Ministry of Public Health, Muang Nonthaburi, Thailand; 7 Laboratory of Management in Public Health, Chisinau, Republic of Moldova; 8 Department of Public Health, Universidad de los Andes, Santiago, Chile; 9 Department of Environmental Health, School of Public Health, Fudan University, Shanghai, China; 10 Department of Environmental Health, University of São Paulo, São Paulo, Brazil; 11 Center for Environmental and Respiratory Health Research, University of Oulu, Oulu, Finland; 12 Medical Research Center Oulu, Oulu University Hospital and University of Oulu, Oulu, Finland; 13 School of Physics, Dublin Institute of Technology, Dublin, Ireland; 14 Institute of Environment, Health and Societies, Brunel University London, London, United Kingdom; 15 Department of Epidemiology, Lazio Regional Health Service, Rome, Italy; 16 Department of Pediatric Infectious Diseases, Institute of Tropical Medicine, Nagasaki University, Nagasaki, Japan; 17 Faculty of Health and Sport Sciences, University of Tsukuba, Tsukuba, Japan; 18 Department of Environmental Engineering, Kyoto University, Kyoto, Japan; 19 Graduate School of Public Health, Seoul National University, Seoul, Republic of Korea; 20 Institute of Environmental Assessment and Water Research, Spanish Council for Scientific Research, Barcelona, Spain; 21 Epidemiology and Environmental Health Joint Research Unit, University of Valencia, Valencia, Spain; 22 Department of Public Health and Clinical Medicine, Umeå University, Umeå, Sweden; 23 National Institute of Environmental Health Sciences, National Health Research Institutes, Zhunan, Taiwan; 24 Department of Environmental and Occupational Medicine, National Taiwan University College of Medicine and National Taiwan University Hospital, Taipei, Taiwan; 25 Department of Environmental Health, Harvard T.H. Chan School of Public Health, Boston, Massachusetts, United States of America; 26 Faculty of Public Health, University of Medicine and Pharmacy, Ho Chi Minh City, Vietnam; 27 Institute of Research and Development, Duy Tan University, Da Nang, Vietnam; 28 School of Forestry and Environmental Studies, Yale University, New Haven, Connecticut, United States of America; 29 Center for Health and the Global Environment, University of Washington, Seattle, Washington, United States of America; 30 School of Public Health and Institute of Environment and Human Health, Anhui Medical University, Hefei, China; 31 Shanghai Children’s Medical Centre, Shanghai Jiao-Tong University, Shanghai, China; 32 School of Public Health and Social Work, Queensland University of Technology, Brisbane, Australia; University of Wisconsin, Madison, UNITED STATES

## Abstract

**Background:**

Heatwaves are a critical public health problem. There will be an increase in the frequency and severity of heatwaves under changing climate. However, evidence about the impacts of climate change on heatwave-related mortality at a global scale is limited.

**Methods and findings:**

We collected historical daily time series of mean temperature and mortality for all causes or nonexternal causes, in periods ranging from January 1, 1984, to December 31, 2015, in 412 communities within 20 countries/regions. We estimated heatwave–mortality associations through a two-stage time series design. Current and future daily mean temperature series were projected under four scenarios of greenhouse gas emissions from 1971–2099, with five general circulation models. We projected excess mortality in relation to heatwaves in the future under each scenario of greenhouse gas emissions, with two assumptions for adaptation (no adaptation and hypothetical adaptation) and three scenarios of population change (high variant, median variant, and low variant). Results show that, if there is no adaptation, heatwave-related excess mortality is expected to increase the most in tropical and subtropical countries/regions (close to the equator), while European countries and the United States will have smaller percent increases in heatwave-related excess mortality. The higher the population variant and the greenhouse gas emissions, the higher the increase of heatwave-related excess mortality in the future. The changes in 2031–2080 compared with 1971–2020 range from approximately 2,000% in Colombia to 150% in Moldova under the highest emission scenario and high-variant population scenario, without any adaptation. If we considered hypothetical adaptation to future climate, under high-variant population scenario and all scenarios of greenhouse gas emissions, the heatwave-related excess mortality is expected to still increase across all the countries/regions except Moldova and Japan. However, the increase would be much smaller than the no adaptation scenario. The simple assumptions with respect to adaptation as follows: no adaptation and hypothetical adaptation results in some uncertainties of projections.

**Conclusions:**

This study provides a comprehensive characterisation of future heatwave-related excess mortality across various regions and under alternative scenarios of greenhouse gas emissions, different assumptions of adaptation, and different scenarios of population change. The projections can help decision makers in planning adaptation and mitigation strategies for climate change.

## Abstract 2

Yuming Guo and colleagues present a modelling study in which climate change-associated mortality is predicted in a large number of countries factoring in different levels of adaptation and greenhouse gas emissions.

## Author summary

**Why was this study done?:**

- Heatwaves can cause a significant impact on population health worldwide, including a rise in mortality and morbidity.
- There will be an increase in the frequency and severity of heatwaves in the future worldwide because of climate change.
- Evidence about the impacts of climate change on heatwave-related mortality at a global scale is limited.

**What did the researchers do and find?:**

- We developed a model to estimate heatwave–mortality associations in 412 communities within 20 countries/regions from January 1, 1984 to December 31, 2015. The associations were used to project heatwave-related excess mortality, with projected daily mean temperature series from four scenarios of greenhouse gas emissions during 1971–2099.
- We used three scenarios of population changes (low, moderate, and high variant) and two adaptation scenarios (no adaptation and hypothetical adaptation).
- If people cannot adapt to future climate change, heatwave-related excess mortality is expected to increase the most in tropical and subtropical countries/regions, while European countries and the United States will have smaller increases. The more serious the greenhouse gas emissions, the higher the heatwave-related excess mortality in the future.
- If people have ability to adapt to future climate change, the heatwave-related excess mortality is expected to still increase in future under the most serious greenhouse gas emissions and high-variant population scenarios. However, the increase is expected to be much smaller than the no adaptation scenario.

**What do these findings mean?:**

- Future changes in heatwave-related excess mortality are highly affected by greenhouse gas emissions and human adaptation to climate change.
- Stricter mitigation policy to reduce greenhouse gas emission is expected to produce greater health benefits related to heatwaves.
- Adaptation strategies should be planned to reduce the health impacts of heatwaves in all the countries/regions, particularly developing countries in tropic and subtropical regions.

## Introduction

A heatwave is often defined as hot outdoor temperature or hot weather that lasts for several days that is outside the normal range of ambient temperatures [1]. Heatwaves can cause heat exhaustion, heat oedema, heat cramps, heat syncope, and heatstroke [2]. They are related to acute cerebrovascular accidents, aggravate chronic pulmonary conditions, cardiac conditions, kidney disorders, and psychiatric illness [3, 4]. Heatwaves can cause a significant impact on population health, including a rise in mortality [5] and morbidity [6]. A number of major heatwave events occurred over the past decade, many of which had devastating effects [7–9]. For example, the European heatwaves in the summer of 2003 were responsible for the deaths of tens of thousands of people [10]. The 2010 Russian heatwaves killed the estimated 55,000 people [11]. Importantly, heatwaves have a greater impact on mortality or morbidity than the reported number of deaths or cases due to classical heat illness (e.g., thermoplegia, heatstroke, heat cramp, and heat syncope), because heatwaves also induce the onset of other diseases, for example, cardiovascular diseases, respiratory diseases, and diabetes. Thus, heatwaves are a critical public health problem.

There will be an increase in the frequency and severity of heatwaves as the globe warms up [12]. An important aspect of understanding the overall risk of climate change for human health is how heatwaves could affect mortality and morbidity under a changing climate [13]. Population vulnerability to heatwaves is strongly influenced by social and physical environments. Thus, the associations between heatwaves and health effects differ by cities, countries, and regions [5]. However, little attention so far has been paid to the projections of heatwave effects across different regions using the same method [14, 15]. To date, most projections of heatwave-related deaths are limited to a few cities/one country or used a small number of climate models [14, 15]. Therefore, the usage of data from different countries and climate zones may help reveal potential spatial heterogeneity in heatwave-related excess mortality and projections.

Our previous study showed the temperatures associated with the lowest mortality averages around the 75th percentile of ambient temperature in all the countries/regions [16]. This percentile of temperature varies slightly by region, suggesting that populations have possibly adapted to some degree to their local climate type. Thus, it is reasonable to expect that people may also have the ability to adapt to increases in the frequency and intensity of heatwaves through adaptation interventions, up to physiological limits. The degree of adaptation to heatwaves is probably explained, in large part, by six levels of adaptation interventions, including individual, interpersonal, community, institutional, environmental, and public policy levels (Table 1). These adaptation interventions could change the human physiology and behaviours affecting the impacts of high temperatures. The details of this process are complex and not completely understood but include physiological change (e.g., rise in core temperature), behavioural changes (e.g., time spent outdoors, clothing, physical activity, healthy lifestyles), improving health services, and environmental improvements (e.g., thermal properties and nature of the built environment including building design and city planning) [3]. Thus, more robust estimates would be generated by accounting for the uncertainties in heatwave adaptation when projecting future heatwave-related excess mortality.

**Table 1:** Selected examples of adaptation interventions, mechanisms, and outcomes at different levels. Adjusted based on reference [17].

Levels of intervention	Methods of intervention	Intervention mechanisms	Intervention outcomes
Individual	Information provision; advertising	Resonance; perceived relevance; reading and reflection	Improving knowledge, and motivation/ intentions; physiological change; behavioural change;
Interpersonal	Information sharing; communication; persuasive arguments; counselling; peer education	Imitating; influence of reference group; mentorship	Improving motivation/intentions; and developing skills/ self-efficacy; behavioural change; indirect influence for physiological change
Community	Strengthening community infrastructure; encouraging community engagement; developing vulnerable people group; livelihoods; neighbourhood watch	Solidarity; diffusion of innovation; changing community norms	Improving motivation/intentions, physical activity and sense of security; behavioural change; indirect influence for physiological change
Institutional	Institutional policies; quality standards; formal procedures and regulations; partnership working	Authorisation; inspection; enforcement; increasing staff awareness	Reducing discrimination and improving services; behavioural change; indirect influence for physiological change
Environmental	Urban planning and management; built environment; planting trees; public available drink water; house quality	Legislation; enforcement; redesign of services; ‘choice architecture’	Environmental improvements; healthier housing; more physical activity; behavioural change; indirect influence for physiological change
Public policy	Improvement of health services; poverty reduction; redistribution of resources; education; heatwave-warning system	Legislation and enforcement; medical accessibility; economic security and choices	Healthy lifestyles; more affordable and given higher priority; behavioural change; indirect influence for physiological change

This study aimed to quantify the excess deaths associated with heatwaves in 412 communities in 20 countries/regions, for the period of 2031–2080 under several global climate change scenarios. An important aspect of this analysis was the partitioning of uncertainties from different sources in the estimation of the excess deaths attributable to heatwaves, including those from statistical variation, climate models, climate change scenarios, adaptation, and population change.

## Methods

### Data collection

#### Historical data on mortality and weather

**Fig 1:**
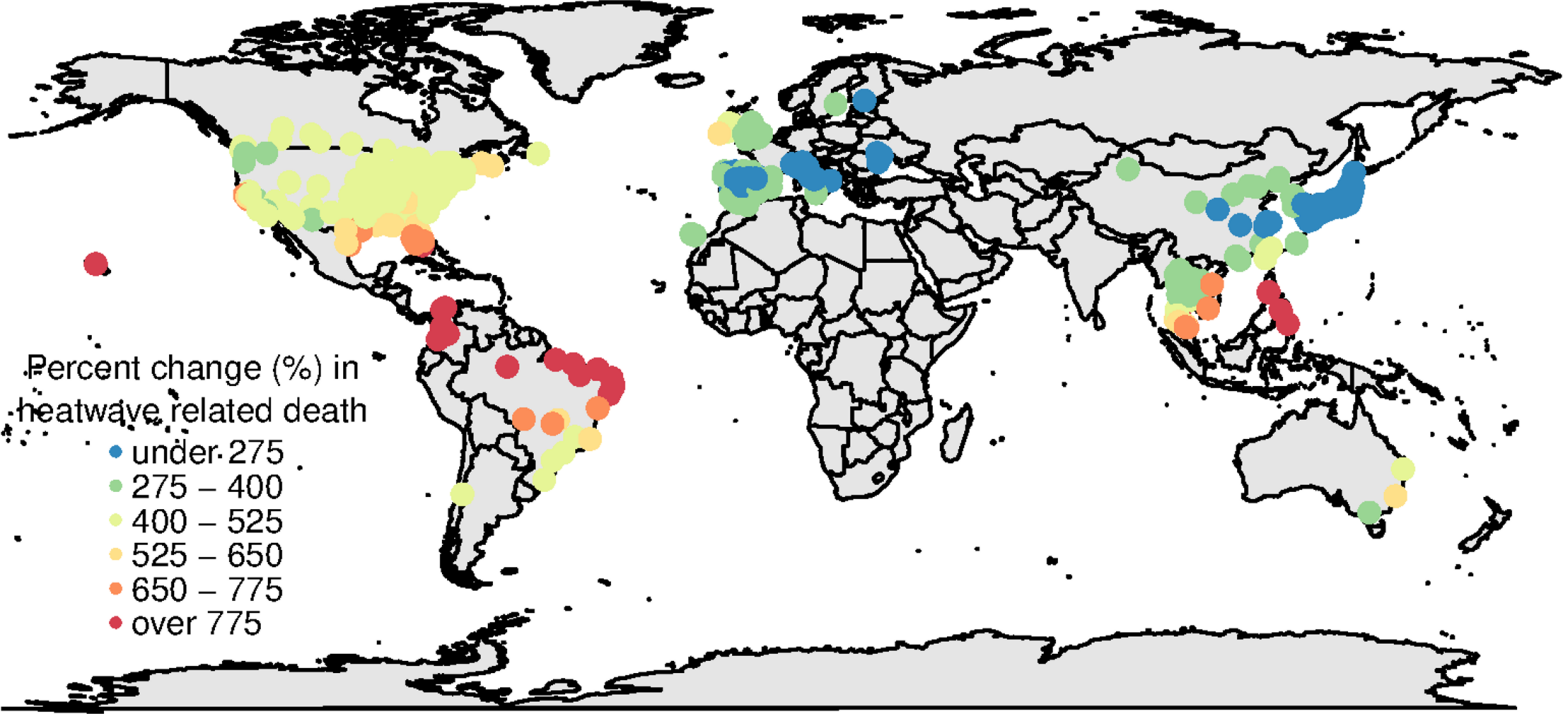
Locations of communities and mean percent change of heatwave-related excess deaths in 2031–2080 comparing to 1971–2020, under RCP8.5 scenario and high-variant population scenario, with assumption of nonadaptation. RCP, Representative Concentration Pathway.

We partly described data collection in our previous publications [5, 16, 18–21]. A detailed description of the data is provided in S1 Appendix. In brief, since 2012, we have developed a Multi-City Multi-Country (MCC) Collaborative Research Network (http://mccstudy.lshtm.ac.uk/) to collect historical data on weather and mortality. In this study, we used daily time series data from 412 communities within 20 countries/regions (**Fig 1**). The study periods overlapped largely, ranging from January 1, 1984, to December 31, 2015 (**Table 2**). The dataset included observed daily time series for deaths counts for all causes or nonexternal causes (International Classification of Diseases [ICD]—ICD-9: 0–799; ICD-10: A00–R99) and daily mean temperature, in addition to weather variables (daily minimum and maximum temperatures and relative humidity). The observed daily mean temperature was measured as the average across 24 h or between maximum and minimum daily temperature from a single or multiple monitoring stations within the administrative boundaries of each community. Community-specific metavariables include weather indices derived from the series of observed temperatures (e.g., average and range of annual, summer, and winter temperatures), climatological zones based the Köppen–Geiger classification, and country-specific gross domestic product (GDP) per capita.

**Table 2:** Summary statistics by country. Temperatures are average community-specific 95th percentile of temperature (heatwave threshold) as GCM ensemble, in the period 1971–2020 and the period 2031–2080. Percent changes of population are changes of population under three population scenarios (low variant, median variant, and high variant) during the period 2031–2080 compared with the period 1971–2020.

County/region	Period of historical data	Number of communities	Total number of deaths	Mean (across communities) of community-specific 95th percentile of daily temperature (°C)					Percent change of population variant between period 2031–2080 and 1971–2020		
				Period 1971–2020	Period 2031–2080						
					RCP2.6	RCP4.5	RCP6.0	RCP8.5	Low	Median	High
Australia	1988–2009	3	1,177,950	25.3	26.0	26.4	26.5	27.2	74.6	98.4	124.8
Brazil	1997–2011	18	3,401,136	28.3	29.3	29.8	29.8	30.8	31.1	52.6	77.5
Canada	1986–2011	26	2,989,901	21.7	22.7	23.3	23.5	24.8	44.5	64.0	85.7
Chile	2004–2014	1	325,462	20.8	21.7	22.2	22.1	23.1	32.7	53.0	76.3
China Mainland	1996–2008	15	950,130	27.4	28.8	29.2	29.3	30.4	1.2	15.9	32.5
Colombia	1998–2013	5	267,736	25.1	25.9	26.4	26.3	27.4	32.9	55.3	81.1
Finland	1994–2011	1	130,325	19.5	20.5	21.2	21.1	22.1	3.7	18.1	34.3
Ireland	1984–2007	6	1,058,215	16.1	17.1	17.5	17.5	18.3	38.0	57.3	78.8
Italy	1987–2010	11	820,390	25.9	27.1	27.6	27.9	29.1	−16.4	−5.7	6.4
Japan	1985–2012	47	26,893,197	27.0	27.9	28.2	28.3	29.2	−22.7	−12.7	−1.5
Moldova	2001–2010	4	59,906	25.0	26.2	26.6	26.8	28.3	−34.6	−23.5	−10.6
Philippines	2006–2010	4	274,516	29.7	30.3	30.7	30.7	31.6	104.6	140.2	180.5
South Korea	1992–2010	7	1 26,938	26.5	27.7	27.8	28.1	29	1.4	14.9	30.0
Spain	1990–2014	51	3,017,110	25.7	26.7	27.1	27.4	28.7	−5.1	7.3	21.2
Sweden	1990–2002	1	190,092	19.2	20.2	20.8	20.9	21.7	20.1	36.7	55.2
Taiwan	1994–2007	3	765,893	28.4	29.2	29.5	29.6	30.4	−2.2	11.6	26.9
Thailand	1999–2008	62	1,827,853	32.4	33.2	33.8	33.8	34.7	−0.1	15.2	33
UK	1990–2012	10	12,075,623	17.8	18.9	19.4	19.5	20.4	14.1	30.1	48
USA	1985–2009	135	22,953,896	26.8	27.8	28.4	28.5	29.7	34.5	54.2	76.2
Vietnam	2009–2013	2	108,173	30.0	31.0	31.5	31.5	32.4	45.1	67.7	93.2

**Abbreviations:** GCM, General Circulation Model; RCP, Representative Concentration Pathway.

#### Projected daily temperature series under climate change scenarios

We obtained climate projections from a database developed under the Inter-Sectoral Impact Model Intercomparison Project (ISIMIP, https://www.isimip.org/), in the form of daily temperature series for historical (1950–2005) and future (2006–2100) periods. The ISIMIP database includes single runs of General Circulation Models (GCMs) developed within the Coupled Model Intercomparison Project Phase 5 (CMIP5). Four greenhouse gas emission scenarios (Representative Concentration Pathway [RCP]2.6, RCP4.5, RCP6.0, and RCP8.5) [22], described in the Fifth Assessment Report (AR5) of the United Nations Intergovernmental Panel on Climate Change (IPCC), were used. Specifically, the ISIMIP Fast Track database provides temperature series for each RCP for five GCMs, namely GFDL-ESM2M, HadGEM2-ES, IPSL-CM5A-LR, MIROC-ESM-CHEM, and NorESM1-M.15. These GCMs are representative of the range of projections of future climate across the CMIP5 models [23]. The model outputs were bias-corrected and downscaled through bi-linear interpolation at a 0.5° × 0.5° spatial resolution and linear interpolated by day of the year [24]. The modelled daily temperature series for each of the 412 communities in the period 1971–2099 were extracted by linking the coordinates with the corresponding grid cells of the climate projections.

The five GCMs reproduced the temperature distribution during the observed period with reasonable accuracy [25]. Differences between the observed and modelled temperature series can occur for several reasons, including different resolution of the data (point sources versus gridded) and poor performance of climate models in areas with sparse information from meteorological stations [26, 27]. Non-negligible deviations between the two sources can produce biased results in the impact projections, in which the modelled temperature series are applied to exposure–response relationships estimated using observed temperature. For this reason, we additionally corrected modelled temperature series using the bias-correction method developed and applied in ISIMIP [24]. This approach produced recalibrated modelled temperature series using the monthly mean and the daily variability around the monthly mean of observed temperature series. This calibration method ensures that the trend (i.e., the warming signal) in the original data is preserved.

#### Population scenarios

We downloaded the population prospects under three fertility scenarios (high variant, medium variant, and low variant) from the UN Population Division during 1950 and 2099, for each country (https://esa.un.org/unpd/wpp/). As the population prospects data is only available for the country level, we assumed that the change of population in each city was the same as its nationwide population prospect trend. Then, we calculated the annual population data for each city for the years 1950–2099.

### Data analysis

#### Heatwave definition

There is no universally accepted definition of a heatwave, but most incorporate notions of intense heat experienced over a period of days [5]. In the present study, relative thresholds based on the community’s recent long-term daily mean temperature were used to define heatwaves, as they describe historic regional acclimatization to temperatures that were normal for a community. Heatwaves in each of the communities were defined as at least two consecutive days with daily mean temperature exceeding the 95th percentile of the year-round daily temperatures of that city from recent data (i.e., the years representing ‘current’ data).

#### Historic heatwave–mortality relationships

We estimated the association between heatwaves and risk of mortality in each community separately based on the years of data within the ‘current’ timeframe. The analyses were limited to the hot season (four hottest months) for each community. The heatwave–mortality association was examined with a two-stage analytic approach previously applied in an analysis of the historical mortality risk associated with heatwaves using a subset of the same dataset [5].

In the first stage, we used a time series Poisson regression model for hot season data to obtain community-specific estimates allowing for over-dispersed death counts. Seasonality was controlled for using a natural cubic spline with 4 degrees of freedom (equally spaced knots) for day of the season. Long-term trend was controlled for using a natural cubic spline with 1 degree of freedom per 10 years. A categorical variable was used to control for the potential confounding effect of day of the week. As the effects of heatwaves on mortality usually appeared immediately and lasted for several days, a natural cubic spline with 4 degrees of freedom was used to capture the distributed lag effect of waves (as a 0–1 variable) over time, up to 10 days [5]. The residual deviance was used to check the model fit, which is a standard method for time series analysis of the associations between air pollution/temperature and mortality [28]. We chose the last year’s data as test data and other years’ data as training data for each city, to cross-validate our model. The cross-validation indicated that our model had a high predictive ability, with correlation of R^2^ = 0.9 between predicted deaths and observed deaths during heatwave days (Fig A in **S1 Appendix**).

In the second stage, we modelled the community-specific effect estimates of the overall cumulative risk ratios (lag 0–10 days) associated with heatwaves using a meta-regression [21]. The meta-regression included a set of meta-predictors to capture part of the heterogeneity across locations (specifically, indicators for country, indicators for climate classification, and indicators for community-specific hot season average and range of temperature). We then derived the best linear unbiased prediction of the overall cumulative association in each community by the meta-regression, expressed as relative risk. This meta-prediction is only performed for the historical data and not for future data.

#### Projection of future heatwave-related excess mortality

Using the daily mean temperature output from each of five GCMs for the timeframe representing ‘future’ years, we calculated the number of heatwaves per year and the total number of heatwave days under a changing climate [14]. We calculated expected excess deaths during heatwave days per year for each set of climate change scenarios (RCP2.6, RCP4.5, RCP6.0, and RCP8.5) and GCMs, from which we can compute the statistics of change. The expected number of excess deaths during a given heatwave period was calculated for each community as follows:

EDHW=N×(RR−1)×HWN

N=POP×MR,

in which EDHW is excess deaths related to heatwaves; N is the average number of deaths on non-heatwave days (calculated from the ‘current’ years); HWN is the number of the future heatwave days; and RR is the relative risk of mortality in relation to heatwaves. POP is annual population, and MR is daily mortality rate (MR) across all non-heatwave days from the historical data for each community. To quantify the overall mortality impact of heatwaves, we computed the annual excess mortality attributable to heatwaves, which is the expected number of deaths in a 1-year period caused by all heatwaves in that year [3]. This summary of health impact incorporates the change in both the rate at which heatwaves occur and the number of heatwaves in the future. We calculated this summary in the two time periods (1971–2020 for present day and 2031–2080 for the future period), summing the excess deaths across all heatwaves and dividing by the total number of years. Then, we calculated the percent change for the period 2031–2080, using the period 1971–2020 as the baseline reference.

### Uncertainty assessment

It is clear that HWN will be affected by the threshold definition of heatwaves in the future. If we assumed humans would not acclimatise/adapt to the warming temperature, the future heatwave threshold should be the same as present absolute temperature threshold in each community. While the actual adaptation mechanisms and timing are unknown, if we assumed human acclimatise/adapt to the warming temperature with the same response relative to the temperature distribution, the future heatwave threshold would be defined by using the same percentile temperature (95th percentile) within its period 2031–2080 in each community. Thus, to better understand the impacts of future heatwaves, we considered these uncertainties on heatwave adaptation by using two assumptions: 1) no adaptation (using current heatwave threshold) and 2) hypothetical adaptation (heatwave threshold was defined by using the future 95th percentile of temperature, reflecting extreme adaptation through six levels of adaptation interventions [Table 1]). We also considered three population scenarios in the future: 1) high variant, 2) median variant, and 3) low variant. We assumed no change in demographic distribution or baseline mortality rate. In other words, our estimates of excess mortality from heatwaves represent the projected impact of climate change and population change, not the combined impact of changes in other characteristics, such as demographic distribution, mortality rate, and socioeconomic factors.

Other sources of uncertainty in the excess deaths are related to the estimate of the exposure-response relationships and the variability in temperature projections between GCMs [21]. These quantities are represented by the variance of the model coefficient and the variability of the five future daily temperature series generated in each RCP, respectively. We quantified this uncertainty by generating 1,000 samples of the coefficients through Monte Carlo simulations, assuming a normal distribution for the estimated coefficients, and then generating results for each of the five GCMs. We reported the results as point estimates, using the average across climate models (GCM ensemble) obtained by the estimated coefficients, and as empirical confidence intervals (eCIs), defined as the 2.5th and 97.5th percentiles of the empirical distribution across coefficients samples and GCMs. These empirical 95% eCI account for both sources of uncertainty. A quantitative comparison of the two components is provided by the ratio between the average standard deviation of the empirical distribution within each GCM and the standard deviation of the average value between GCMs.

## Results

We analysed multicountry data for 412 communities within 20 countries/regions (**Table 2**). It covers nine regions characterised by different climatic conditions: North America, Central America, South America, northern Europe, central Europe, southern Europe, East Asia, Southeast Asia, and Oceania. The dataset included 79,287,540 deaths observed within overlapping current periods. The 95th percentile of temperature is projected to increase the most in 2031–2080 under RCP8.5, while a small increase is estimated under RCP2.6. Similar patterns are projected for the mean of daily temperature (Table A in **S1 Appendix**). Population will increase under the high-variant scenario in all countries except Japan and Moldova (**Table 2**). Most cities had significant relative risks of mortality associated with heatwaves (Table B in **S1 Appendix**).

**Fig 1** shows the percent change of heatwave-related excess mortality in the period 2031–2080 compared to the period 1971–2020 (representing the present day), under RCP8.5 and the high-variant population scenario, without any adaptation. The change in future heatwave-related excess mortality varies by community. But there is a clear pattern that communities close to the equator or located in tropical or subtropical climates are projected to have a large increase. Communities located in temperate regions are projected to experience a relatively small increase. The results from different population scenarios (high variant, median variant, and low variant) and different greenhouse gas emission scenarios (RCP2.6, RCP4.5, RCP6.0, and RCP8.5) show similar spatial patterns (Fig B-L in **S1 Appendix**). But the magnitude of the change varies by scenario.

**Fig 2:**
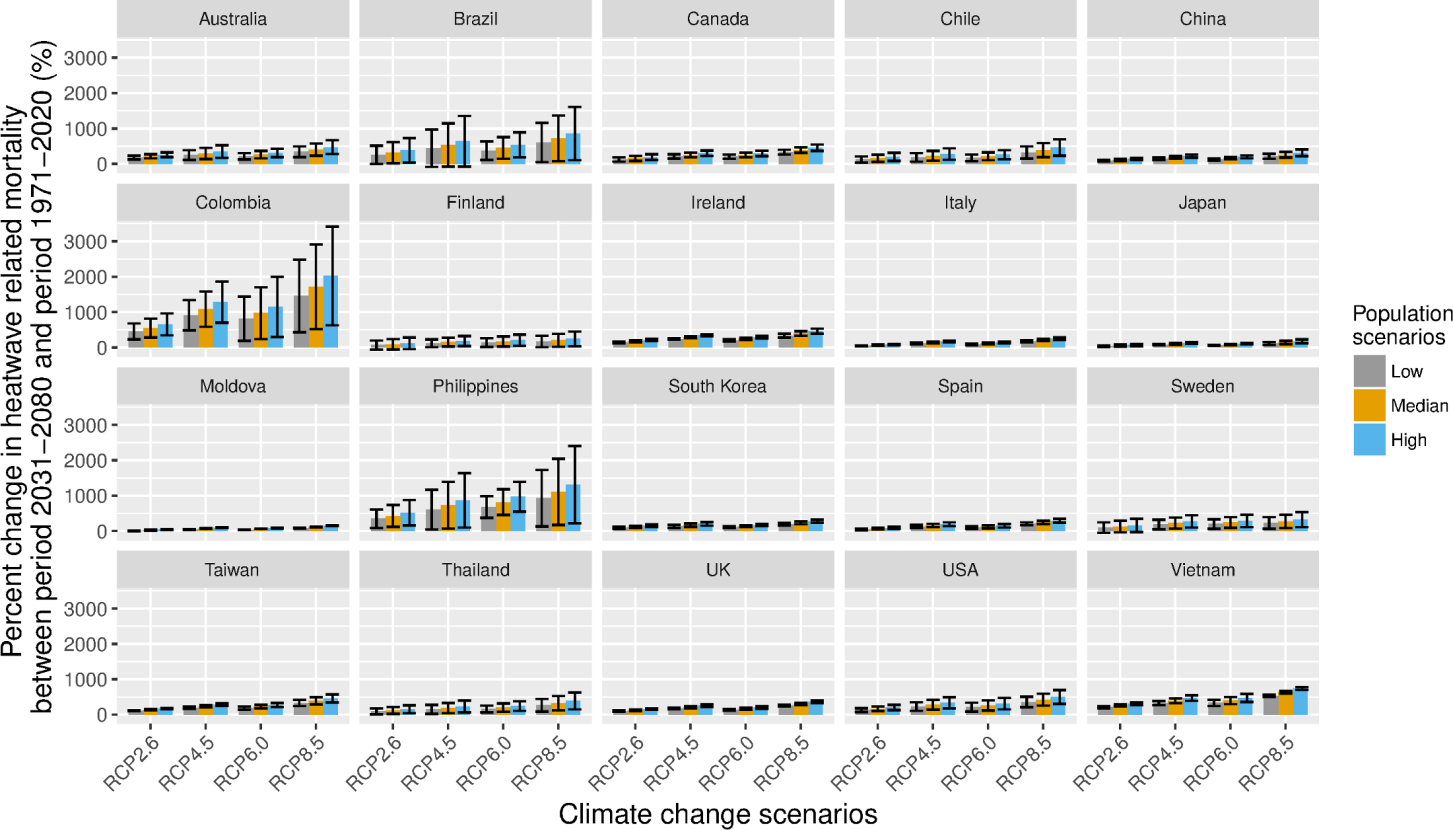
Mean percent change heatwave-related excess deaths in 2031–2080 in comparison to 1971–2020, in 20 countries/regions under RCP2.6, RCP4.5, RCP6.0, and RCP8.5 scenarios and high-variant, median-variant, and low-variant population scenarios, with assumption of nonadaptation. The high–low line indicates 95% eCI. Please refer to Table C in **S1 Appendix** for effect estimates. eCI, empirical confidence interval; RCP, Representative Concentration Pathway.

If we didn’t consider any adaptation, heatwave-related excess mortality is expected to increase the most in tropical and subtropical countries/regions, with the highest increase in Colombia, followed by Philippines and Brazil (**Fig 2**). European countries and the US are expected to have smaller percent increases. For all the countries, RCP8.5 will have a higher change than the other three RCPs in the same period. For the same RCP, the high-variant population scenario produce higher increases than the low-variant and median-variant population scenarios. The details for the effect estimates and 95% eCIs are shown in Table C in **S1 Appendix**. Table E in **S1 Appendix** shows the predicted annual average deaths related to heatwaves in the period 1971–2020.

**Fig 3:**
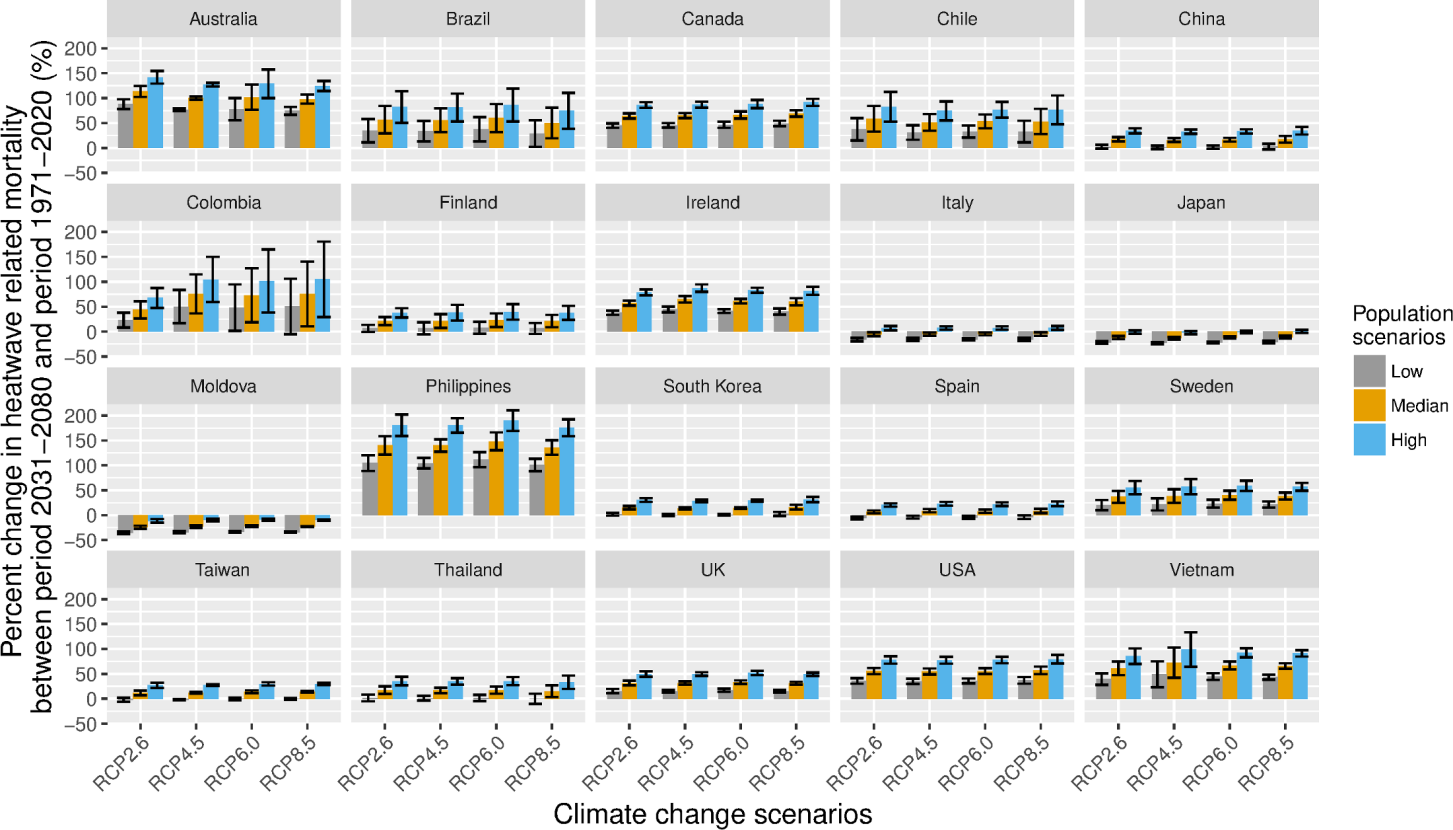
Mean percent change heatwave-related excess deaths in 2031–2080 in comparison to 1971–2020, in 20 countries/regions under RCP2.6, RCP4.5, RCP6.0, and RCP8.5 scenarios and high-variant, median-variant, and low-variant population scenarios, with assumption of full adaptation. The high–low line indicates 95% eCI. Please refer to Table D in **S1 Appendix** for effect estimates. eCI, empirical confidence interval; RCP, Representative Concentration Pathway.

If we considered hypothetical adaptation to the 95th percentile temperature, the increases are much lower than those without accounting for adaptation (**Fig 3** and Table D in **S1 Appendix**). For the high-variant population scenario, the heatwave-related excess mortality is expected to increase in all the countries/regions except Moldova and Japan, under all scenarios of greenhouse gas emissions. For the low-variant population scenario, heatwave-related excess mortality is expected to decrease in Italy, Japan, Moldova, and Spain, under all the scenarios of greenhouse gas emissions. The decreases in heatwave-related excess mortality in Japan and Moldova are mainly driven by population decrease in the future (**Table 2**). The results for each community are similar to the country/region level results (Fig M-X in **S1 Appendix**).

## Discussion

We estimated future changes in heatwave-related excess mortality for 412 communities in 20 countries/regions, using five GCMs and four RCPs. To the best of our knowledge, this study is by far the largest international study for the potential health impacts associated with heatwaves under climate change scenarios. The assessment includes and compares results from hundreds of locations across regions of the world, characterised by different climates, socioeconomic and demographic conditions, and levels of development of infrastructures and public health services. The analysis applies advanced analytical methods to project heatwave-related mortality under different ranges of temperature increase consistent with the RCPs and population change scenarios and accounts for several types of uncertainties. Our results showed that with no adaptation to warmer temperatures, the changes in heatwave-related excess mortality differ substantially across countries/regions. Countries/regions near the equator are projected to experience higher percentage increases in heatwave-related excess mortality under future climate scenarios than those in temperate climate zones. Under the hypothetical adaptation scenarios based on relative temperature, most of heatwave-related excess mortality would be offset in the future, but it is expected to still increase in all countries/regions under high-variant population scenarios except Moldova and Japan, where populations will decrease in the future. However, there is no clear evidence that such hypothetical adaptation would actually happen and be effective and timely, resulting in adverse health outcomes from heatwaves over this century.

The projections indicate strong geographical variability. Some tropical and subtropical areas such as Brazil, Colombia, and Philippines are characterised by a relatively high projected warming and increase in heatwave-related excess mortality. By contrast, all the European countries and the US are projected to experience a small increase in heatwave-related excess mortality. The results are comparable with studies performed in the US [29], Europe [30], China [31], and Korea [32]. Notably, arid or equatorial regions that include a large proportion of the current and projected global population will contribute greatly to the global impact of climate change.

Changes in heatwave-related excess mortality are also highly affected by the extent of warming under different RCPs. The strongest increase in heatwave-related excess mortality is projected under RCP8.5, which is characterised by unabated greenhouse gas emissions, leading to a steep increase in temperature. Conversely, the effects of climate change, and particularly the increase in heatwave-related excess mortality in all countries/regions, are significantly smaller in scenarios assuming mitigation strategies and null or marginally negative under the stricter RCP2.6. These findings highlight the importance of implementation of effective climate policies to minimize increases in ambient temperatures and prevent the associated negative impacts on human health.

Individuals have adapted to and will likely continue to adapt to local climate change, including increasing ambient temperature [16, 29] through six levels of adaptation interventions (Table 1), but only within certain limits [33]. These adaptation interventions could result in a lower rise in core temperature and a lower increase in heart rate at a given heat load [34]. Our findings show that, when we consider hypothetical adaptation related to heatwave thresholds (based on relative temperature), heatwave-related excess mortality would level off for all countries/regions, with a lowered expected heatwave-related excess mortality. This means ignoring adaptation in projections would result in a substantial overestimate of future heatwave-related excess mortality. In addition, there will be small differences in heatwave-related excess mortality between RCPs. However, this hypothetical adaptation may be quite difficult to achieve in the short-term, because it depends on many factors (Table 1), including the ability of individuals, society, and populations to make modifications within a timeframe adequate to keep pace with changing temperatures. Furthermore, the ability of populations to adapt will differ dramatically by country and by subpopulations.

We have to highlight that we only considered a change of the heatwave threshold as a possible adaptation. We did not consider the change of heatwave-related relative risks (reflecting increasing resilience and vulnerability) affected by behavioural change, improvement of physical activity, healthier housing, and environmental changes through six levels of adaptation interventions, which also indirectly affect the process of physiological change (Table 1) [35]. Incorporating this into models requires projections on adaptation interventions including prevalence of air conditioning, improvement in cooling technology over time, improvement in healthcare systems against heat-related diseases, and improvement in heatwave warning systems [29]. In addition, we also didn’t consider the changes in heatwave intensity (mean temperature during heatwaves) and summer average temperature [36]. With our historical data, we found that higher heatwave intensity was associated with higher heatwave-related mortality risk, while higher summer average temperature was related to lower heatwave-related mortality risk (Fig Y in **S1 Appendix**). As both heatwave intensity and average summer temperature will increase in the future, the change in relative risks of mortality related to heatwaves would be balanced by these two factors. Further research is needed to better understand the mechanisms of adaptation interventions with the goal of reducing the detrimental health impacts of future heatwaves.

To estimate future heatwave-related excess mortality, we relied on many assumptions. Multiple climate model simulations of future climate change were used to account for variation in climate models’ structural assumptions, an important source of uncertainty in future projections [14]. Intermodel variability is significant even at global average scales, but it becomes increasingly relevant as the output of global models is used to describe climate change at small regional scales and for high-frequency quantities like daily output, as in the case of our analysis. In addition, we used meta-regression with historical data to project historical community-specific heatwave–mortality relative risks to project future heatwave-related excess mortality in that community. Although this method cannot solve the problem that future relative risks might be different than those in the past, this can eliminate the potential between-city differences when applying historical data from a warmer city to predict the future mortality of another warming city [37].

There is a problem in relation to projections of daily temperatures at local scales. The downscaling methods assume future variation/trend of daily temperature are the same as current. This assumption may be appropriate for the near future, but the situation is uncertain for more distant future climates. Although there are good estimates of future climates from GCMs, it is clear that present and future predictability of temperature variability is not the same everywhere [38]. It is difficult to evaluate the adequacy of different downscaling methods, because there is limited information on how climate change affects local-level daily weather. Importantly, there is a significant gap between the available information at seasonal time scales and the information at longer time scales. Information about what is likely over the next decades is largely unknown [39]. This highlights the importance of using different scenarios and different models to assess possible future daily temperatures and their impacts.

Our findings are consistent with previous studies in single locations or countries for results considering no adaptation [30–32] and adaptation [29]. Most findings were limited to a no adaptation assumption and reported large increases in future heatwave-related excess mortality. A study projected that Chicago is expected to have 166–2,217 heatwave-related excess deaths per year during 2081–2100, based on estimates from seven global climate models under three different climate change scenarios, without any adaptation. The largest cause of variation in the projections was the choice of climate model [14]. A recent study conducted in the US estimated that, accounting for adaptation, the overall heat-related mortality by 2050 would not change substantially compared to 2006 [29]. In particular, the variety of analytical designs, with alternative effect summaries, statistical modelling, and assumptions, makes it difficult to quantitatively compare results and to draw a comprehensive picture of the global impact of climate change directly attributable to changes in heatwaves. By contrast, our assessment applies an advanced and well-tested statistical framework uniformly across various counties/regions and climates, accounting for community-specific heatwave–mortality relationships, and provides a consistent overview of geographical and temporal differences.

Some limitations have to be acknowledged. Our projections of heatwave–related mortality associations under five greenhouse gas emission scenarios only include simple assumptions with respect to adaptation: no adaptation and hypothetical adaptation. Our assessment of adaptation does not comprehensively address the complexities of this issue, including the timing of adaptive measures, effectiveness, existence of prerequisites for implementing adaptation options, etc. This work considered the number of heatwaves and heatwave days, although previous research indicates that the intensity of heatwaves also impacts health response. Should heatwaves be more intense in the future, even if the number of heatwave days stays the same, an increase in adverse health outcomes would be anticipated. The method we used to define heatwaves allows for regional acclimatization to temperatures normal for a community, but this would possibly make every community have heatwaves. The findings should therefore be interpreted as potential impacts under hypothetical scenarios and not as projections of future excess mortality. This study does not provide evidence for large areas of the world owing to insufficient data. Estimates are also affected by considerable uncertainty, due to variability in the climate models, population change scenarios, and imprecision in the estimated heatwave–mortality associations. The heatwave–mortality association is often larger and mainly related to uncertainty in applying current associations to the future. This would result in an underestimation of heatwave-related excess deaths. As there were limited data for verification of models in some tropical countries, the projections for those places have larger uncertainties. While we generated community-specific estimates of heatwaves and mortality, the health response may differ by subpopulation within a community. We did not consider the influence of climate–urban heat island (UHI) interaction on heatwave–mortality associations. However, its influence is much smaller than adaptation, mitigation, and population change, and the data on climate–UHI interactions are not available. Future work could consider these population differences, as well as how demographic shifts, urbanization patterns, and population migration/dynamics could affect heatwave–mortality risk from climate change (e.g., larger effects for older populations in conjunction with an aging population).

In summary, the uncertainties of climate change and adaptation assumptions are not assigned probabilities but rather can be deemed as possible futures, which depend on demographic, technological, political, social, and economic developments [40]. Informed by quantitative or qualitative evidence, projections provide decision makers with information on a range of possible future trends, contexts, risks, and opportunities. Our findings can provide suggestions for real-world practices for climate change mitigation and adaptation. First, stricter mitigation policy has to be applied to reduce greenhouse gas emission, because lower greenhouse gas emissions are associated with fewer deaths due to heatwaves. Second, adaptation interventions should be planned to reduce the health impacts of heatwaves in all the countries/regions, particularly for developing countries in tropical and subtropical regions. The adaptation interventions include six levels (individual, interpersonal, community, institutional, environmental, and public policy levels) with many methods, for example, communicating the health risks of heatwaves to the public and policy makers, establishing early warning systems and urban cooling centres, and developing smart house technology (cost effective to keep cool in summer) (Table 1).

## Supporting information

S1 Appendix Data collection and supporting tables and figures. (PDF)

## Abbreviations

AR5: Fifth Assessment ReportCMIP5, Coupled Model Intercomparison Project Phase 5
eCI: empirical confidence interval
GCM: General Circulation Model
GDP: gross domestic product
ICD: International Classification of Diseases
IPCC: Intergovernmental Panel on Climate Change
ISIMIP: Inter-Sectoral Impact Model Intercomparison Project
MCC: Multi-City Multi-Country
MR: mortality rate
RCP: Representative Concentration Pathway
UHI: urban heat island
