# Supplementary material for: Quantifying excess deaths related to heatwaves under climate change scenarios: A multicountry time series modelling study
Source: PLoS Med. 2018 Jul 31;15(7):e1002629. doi: 10.1371/journal.pmed.1002629 (PMC6067704; doi:10.1371/journal.pmed.1002629)
Supplement: S1 Appendix — (PDF) [file pmed.1002629.s001.pdf]

S1 Appendix

Quantifying excess deaths related to heatwaves under climate scenarios: A multi-country time-series modelling study

Data collection

Australia

We collected data from Melbourne, Sydney and Brisbane between 1st of January 1988 and 31st of May 2009. Daily mortality, obtained from the Australian Bureau of Statistics, is represented by counts of deaths for non-external causes only (ICD-9: 0-799; ICD-10: A00-R99). Daily minimum, mean (24-hour average) and maximum temperature (in ˚C) and relative humidity (in %) were obtained from the Australian Bureau of Meteorology. We selected all available meteorological stations located within ≤30 km of each city's Central Business District (CBD) (7 stations in Brisbane, 7 stations in Melbourne and 11 stations in Sydney). We calculated the daily averages of climatic variables using all records from meteorological stations in each city. When there was a missing value (≤1.3%) for a particular meteorological station, observations recorded from the remaining weather stations were used to compute the daily average values.

Brazil

Data on daily deaths for non-external causes only (ICD-10 codes: A00-R99) in 18 cities (see full list in Table S1 below) between 1st of January 1997 and 31st of December 2011 were collected from Ministry of Health, Brazil. Data on mean daily temperature (computed as the 24-hours average based on hourly measurements) and relative humidity were obtained during the same study period from Weather Meteorological Service of Brazil.

Canada

We obtained daily data on non-accidental mortality from Statistics Canada through access to the Canadian Mortality Database for the period of 1986 to 2009 for 21 census metropolitan areas (CMA). Daily meteorological data were obtained from Environment Canada using the

airport monitoring station located closest to the CMA centre. Daily averages of temperature and relative humidity were computed based on hourly measurements.

Chile

We collected data from the city of Santiago de Chile between 1st of January 2008 and 31st of December 2014. Daily mortality, obtained from Departamento de Estadísticas e Información de Salud (Ministerio de Salud), is represented by counts of deaths for all causes. Mean daily temperature (in ˚C), computed as 24-hour average based on hourly measurements, were obtained from Sistema de Información Nacional de Calidad del Aire (SINCA), Ministerio del Medio Ambiente. A single weather station (Parque Ohiggins) was selected. In total, missing data amount for 0·00% and 1·17% of the mortality and temperature series, respectively.

China Mainland

We collected data from the city of Anshan (1st of January 2004 to 31st of December 2006), Beijing (1st of January 2007 to 30th of September 2008), Fuzhou (1st of January 2004 to 31st of December 2006), Guangzhou (1st of January 2007 to 31st of December 2008), Hong Kong (1st of January 1996 to 31st of December 2002), Hangzhou (1st of January 2002 to 29th of December 2004), Lanzhou (1st of January 2004 to 31st of December 2008), Nanjing (1st of January 2007 to 31st of December 2010), Shanghai (1st of January 2001 to 31st of December 2004), Shenyang (1st of January 2005 to 31st of December 2008), Suzhu (1st of January 2005 to 31st of December 2008), Taiyuan (1st of January 2004 to 31st of December 2008), Tianjin(1st of January 2005 to 31st of December 2008), Wuhan (1st of January 2003 to 31st of December 2005), Wulumqi (1st of January 2006 to 31st of December 2007), and Xian (1st of January 2004 to 31st of December 2008). Daily mortality is represented by counts of deaths for non-external causes only (ICD-9: 0-799; ICD-10: A00-R99). Mean daily temperature (in

˚C) and relative humidity (in %) were computed as the 24-hour average based on hourly measurements. Measures of nitrogen dioxide (NO2, in µg/m^3), particles (PM10, in µg/m^3) and sulphur dioxide (SO2, in µg/m^3) were available in the same period. Daily level of pollutants were computed as the 24-hour mean based on hourly measurements. In total, missing data amount for 0.54% and 0.33% of the mortality and temperature series, respectively

Colombia

We gathered daily data from the 5 biggest Colombian cities (Bogotá, Cali, Medellin, Barranquilla and Cartagena), from 1998 to 2013. Meteorological data including daily maximum, mean, minimum temperatures (Celsius grades), and mean relative humidity (%) were gathered from one station at each city. Daily non-accidental mortality data were obtained from each city's Statistics Department during the same study period.

Finland

We collected data from the city of Helsinki between the 1st of January 1994 and 31st of December 2011. Daily mortality, obtained from Statistics Finland, is represented by counts of deaths for all causes. A dataset containing minimum, mean, and maximum daily temperatures was obtained from the Finnish Meteorological Institute. In this dataset, point measurements from the weather measuring stations around the country have been interpolated onto a 10×10 km grid covering the whole of Finland, using a Kriging model. The temperature variables in the Helsinki Temperature Time-series have been extracted from the GIS-database for KKJ-coordinates 6675470:2552920 (KKJ, Finnish National Coordinate System based on ED50). These are the coordinates for weather measuring station Kallion urheilukenttä of Helsinki Region Environmental Services Authority HSY.

Ireland

Daily non-accidental deaths were obtained from the Irish Central Statistics Office for data in the republic of Ireland (ROI), and Northern Ireland Social Research Agency for data in Northern Ireland (NI) for the period of January 1st 1984 and December 31st 2007. Daily time-series weather data for the study period were obtained from Met Eireann, the Irish Meteorological Service, for ten weather stations in the ROI: Birr, Clones, Casement Aerodrome, Cork, Dublin, Kilkenny, Malin Head, Rosslare, Shannon and Valentia. The weather for NI was obtained from the United Kingdom Meteorological Office for four weather stations with full time-series data: Aldergrove, Armagh, Ballywatticock, and Banagher. The data included daily maximum, minimum, and mean temperatures, relative humidity and air pressure.

Italy

We obtained daily data on mortality from all causes among the resident population dying within the city for Palermo, Bari, Latina, Frosinone, Roma, Viterbo, Bologna and Brescia; no-accidental causes were collected for Genova and Torino. Data were extracted from local mortality registries and from the rapid mortality surveillance system operational since 2004. Meteorological data referring to the airport station located closest to the city centre were obtained from the Meteorological Service of the Italian Air Force.

Japan

Data on daily deaths for non-external causes only (ICD-9 codes: 1-799; ICD-10 codes: A00-R99) in 47 prefectures (see full list in Table S1 below) between 1st of January 1985 and 31st of December 2012 were collected. Data on daily minimum, mean (computed as the 24-hours average based on hourly measurements) and maximum temperatures and relative humidity were obtained for the same study period.

Moldova

Data on daily deaths for non-external causes only (ICD-10 codes: A00-R99) in 4 cities (see full list in Table S1 below) between 1st of January 2001 and 31st of December 2010 were collected from National Centre of Public Health Management. Data on daily minimum, mean (computed as the 24-hours average based on hourly measurements) and maximum temperatures and relative humidity were obtained by State Hydrometeorological Service for the same study period.

Philippines

Data on daily deaths for non-external causes only (ICD-10 codes: A00-R99) in 4 cities (see full list in Table S1 below) between 1st of January 2006 and 31st of December 2010 were collected from the Philippine Statistics Authority. Data on daily minimum, mean (computed as the 24-hours average based on hourly measurements) and maximum temperatures and relative humidity were obtained by the Philippine Atmospheric, Geophysical and Astronomical Services Administration (PAGASA) for the same study period.

South Korea

Data on daily deaths for non-external causes only (ICD-9 codes: 1-799; ICD-10 codes: A00-R99) in 7 cities (see full list in Table S1 below) between 1st of January 1992 and 31st of December 2010 were collected. Data on daily minimum, mean (computed as the 24-hours average based on hourly measurements) and maximum temperatures and relative humidity were obtained for the same study period.

Spain

We obtained daily data on non-accidental causes for the 51 capital cities from the Spain National Institute of Statistics for summer months (from 1st June to 30th September) from 1990 to 2014. Daily minimum, mean and maximum temperatures for the 51 capital cities were collected from the Spain National Meteorology Agency for the same study period. We did not get the data on relative humidity, because it is not available.

Sweden

We collected data from the county of Stockholm between 1st of January 1990 and 31st of December 2002. Daily mortality, obtained from the Swedish Cause of Death Register at the Swedish National Board of Health and Welfare, is represented by counts of deaths for non-external causes only (ICD-9: 0-799; ICD-10: A00-R99). Mean daily temperature (in ˚C) and relative humidity (in %), computed as the 24-hour average based on hourly measurements, were obtained from the Environment and Health Administration. A single weather station, located at Torkel Knutssongatan in Central Stockholm, was selected. Measures ozone (O3, in ppb) and nitrogen oxides (NOx, in ppb) were available in the same period. Daily level of pollutants were computed as the 24-hour mean based on hourly measurements. In total, missing data amount for 0.00% and 6.59% of the mortality and temperature series, respectively. These data were used and described in previous publications.

Taiwan

Data on daily deaths for non-external causes (ICD-9 codes: 1-799; ICD-10 codes: A00-R99) in Kaohsiung, Taipei and Taichung between 1st of January 1994 and 31st of December 2007 were collected. Data on daily minimum, mean (computed as the 24-hours average based on hourly measurements) and maximum temperatures and relative humidity were obtained for the same study period.

Thailand

We obtained daily data on non-accidental deaths from the Ministry of Public Health, Thailand for 62 provinces during 1999–2008. The daily weather data (daily minimum, mean, and maximum temperatures and mean relative humidity) were obtained from the Meteorological Department, Ministry of Information and Communication Technology. There were 117 weather stations in 62 provinces, with at least one weather monitoring station in each province.

UK

We obtained daily data on non-accidental mortality from the Office of National Statistics during 1993–2006. Records include the date of death and postcode of residence at time of death. The postcodes were used to divide deaths into 10 government regions and date to make daily series of counts for each region. The daily weather data (daily minimum, mean, and maximum temperatures and mean relative humidity) were downloaded from the British Atmospheric Data Centre. There was a mean of 29 stations contributing data to each regional series, from a minimum of 7 in London to a maximum of 44 in Wales.

USA

We collected data from 135 cities (see full list in Table S1) between 1st of January 1985 and 31st of December 2009. Daily mortality, obtained from the National Center for Health Statistics (NCHS), is represented by counts of deaths for non-external causes only (ICD-9: 0-799; ICD-10: A00-R99). Daily minimum, mean (in ˚C, computed as the 24-hour average based on hourly measurements) and maximum temperatures and relative humidity (in %, computed from the 24-h average of hourly measurements of dew point temperature) were obtained from the National Climatic Data Center (NCDC) of the National Oceanic and Atmospheric

Administration (NOAA). A single weather station was selected for each city in the land based station data or NCDC, based on the proximity to the city's population centre. In 6 cities where multiple observations were missing from all the nearby monitors, hourly data from the Integrated Surface Database Lite of NCDC were converted in daily values. For 25 stations missing dew point data, dew point data were obtained from the nearest station with dew point data.

Vietnam

Data on daily deaths for non-external causes only (ICD-10 codes: A00-R99) in 2 cities (Ho Chi Minh City and Hue) between 1st of January 2009 and 31st of December 2013 were collected from the A6 mortality reporting system, Vietnam. Data on daily minimum, mean (computed as the 24-hours average based on hourly measurements) and maximum temperatures and relative humidity were obtained by the US National Oceanic and Atmospheric Administration's National Climate Data Center for the same study period.

Table A: Average community-specific mean of temperature as GCM-ensemble in different periods by country/region.

County/Region	Mean (across communities) of community-specific mean of daily temperature (°C)				
	Period 1971-2020	Period 2031-2080			
		RCP2.6	RCP4.5	RCP6.0	RCP8.5
Australia	18.0	18.6	19	19	19.7
Brazil	25.1	26	26.3	26.4	27.1
Canada	7.0	8.2	8.5	8.8	9.8
Chile	13.1	13.8	14.3	14.2	15.0
China	14.2	15.4	15.7	15.9	16.8
Colombia	23.5	24.3	24.6	24.6	25.4
Finland	5.4	7.1	7.7	7.9	8.7
Ireland	9.2	10.2	10.5	10.5	11.0
Italy	15.1	16.2	16.5	16.6	17.4
Japan	14.4	15.5	15.7	15.9	16.7
Moldova	11.1	12.4	12.8	12.9	13.7
Philippines	27.2	28	28.4	28.4	29
South Korea	12.8	14.1	14.2	14.5	15.3
Spain	15.1	16.2	16.4	16.6	17.4
Sweden	7.1	8.6	9.0	9.2	9.8
Taiwan	22.0	22.9	23.1	23.2	23.8
Thailand	28.0	29	29.3	29.4	30.1
UK	9.8	10.6	10.9	11	11.5
USA	14.7	15.9	16.2	16.5	17.3
Vietnam	26.5	27.4	27.7	27.8	28.5

Table B: The relative risks (and 95% CI) of mortality associated with heatwaves in 412 cities.

Country	City	RR	95% CI	
			Low	High
Australia	Brisbane	1.04	0.97	1.12
Australia	Melbourne	1.11	1.05	1.18
Australia	Sydney	1.08	1.03	1.13
Brazil	Belem	1.08	1.04	1.13
Brazil	Belo Horizonte	1.12	1.08	1.17
Brazil	Brasilia	1.05	1.01	1.1
Brazil	Cuiaba	1.07	1.02	1.12
Brazil	Curitiba	1.05	1.01	1.1
Brazil	Fortaleza	1.08	1.04	1.13
Brazil	Goiania	1.08	1.04	1.12
Brazil	Joao Pessoa	1.07	1.02	1.12
Brazil	Maceio	1.05	1.01	1.1
Brazil	Manaus	1.08	1.04	1.13
Brazil	Natal	1.06	1.01	1.1
Brazil	Porto Alegre	1.15	1.1	1.19
Brazil	Recife	1.07	1.03	1.11
Brazil	Salvador	1.07	1.03	1.11
Brazil	Sao Luis	1.07	1.02	1.12
Brazil	Sao Paulo	1.12	1.08	1.16
Brazil	Teresina	1.09	1.04	1.14
Brazil	Vitoria	1.08	1.04	1.13
Canada	Abbotsford	1.06	1.01	1.11
Canada	Calgary	1.04	1	1.08
Canada	Edmonton	1.04	1.01	1.08
Canada	Halifax	1.05	1.01	1.09
Canada	Hamilton	1.04	1	1.08
Canada	Kingston	1.04	1	1.09
Canada	Kitchener-Waterloo	1.06	1.02	1.11
Canada	London Ontario	1.06	1.02	1.1
Canada	Montreal	1.13	1.09	1.17
Canada	Ottawa	1.03	0.99	1.07
Canada	Regina	1.05	1.01	1.09
Canada	Saint John NB	1.02	0.98	1.06
Canada	Saskatoon	1.04	1	1.09
Canada	St. John's NFL	1.05	1	1.09
Canada	Sudbury	1.03	0.99	1.07
Canada	Thunder Bay	1.04	1	1.09
Canada	Toronto	1.07	1.04	1.1
Canada	Vancouver	1.06	1.02	1.09
Canada	Victoria	1.05	1.01	1.1
Canada	Windsor	1.04	0.99	1.09
Canada	Winnipeg	1.03	0.99	1.06

Canada	Niagara	1.07	1.02	1.11
Canada	Oakville	1.04	1	1.09
Canada	Oshawa	1.06	1.02	1.11
Canada	Sarnia	1.04	1	1.09
Canada	Sault Ste. Marie	1.06	1.02	1.1
Chile	Santiago	1.05	0.98	1.14
China	Anshan	1.1	1.05	1.15
China	Beijing	1.1	1.05	1.15
China	Fuzhou	1.12	1.06	1.18
China	Guangzhou	1.17	1.11	1.23
China	Hangzhou	1.13	1.08	1.19
China	Hong Kong	1.09	1.04	1.14
China	Lanzhou	1.02	0.95	1.11
China	Shanghai	1.15	1.11	1.2
China	Shenyang	1.09	1.04	1.15
China	Suzhu	1.15	1.09	1.2
China	Taiyuan	1.11	1.05	1.17
China	Tianjin	1.1	1.05	1.16
China	Wuhan	1.2	1.14	1.26
China	Wulumqi	1.09	1.03	1.16
China	Xian	1.11	1.05	1.16
Colombia	Barranquilla	1.05	0.98	1.12
Colombia	Bogota	0.99	0.93	1.06
Colombia	Cali	1.07	1.01	1.13
Colombia	Cartagena	1.06	0.99	1.14
Colombia	Medellin	1.04	0.98	1.1
Finland	Helsinki	1.13	1.05	1.22
Ireland	Aldergrove and Armagh	1.03	1	1.07
Ireland	Ballywatticock and Banagher	1	0.97	1.05
Ireland	Birr and Kilkenny	1.04	0.99	1.08
Ireland	Dublin and Casement	1.01	0.97	1.05
Ireland	Malin and Clones	1	0.96	1.05
Ireland	Roslare, Cork, Valentia and Shannon	1.05	1.01	1.09
Italy	Bari	1.4	1.33	1.47
Italy	Bologna	1.43	1.37	1.51
Italy	Genoa	1.33	1.27	1.39
Italy	Palermo	1.43	1.35	1.51
Italy	Rome	1.35	1.3	1.4
Italy	Turin	1.32	1.26	1.38
Italy	Brescia	1.4	1.33	1.47
Italy	Civitavecchia	1.39	1.31	1.46
Italy	Frosinone	1.38	1.31	1.45
Italy	Latina	1.39	1.32	1.46
Italy	Viterbo	1.37	1.3	1.44
Japan	Fukuoka	1.04	1.02	1.06
Japan	Osaka	1.06	1.03	1.08

Japan	Tokyo	1.09	1.06	1.11
Japan	Aichi	1.09	1.07	1.11
Japan	Akita	1.07	1.04	1.1
Japan	Aomori	1.12	1.08	1.15
Japan	Chiba	1.03	1.01	1.05
Japan	Ehime	1.05	1.03	1.08
Japan	Fukui	1.03	1	1.06
Japan	Fukushima	1.05	1.03	1.08
Japan	Gifu	1.08	1.05	1.1
Japan	Gunma	1.05	1.03	1.08
Japan	Hiroshima	1.05	1.02	1.07
Japan	Hokkaido	1.05	1.02	1.08
Japan	Hyogo	1.03	1.01	1.05
Japan	Ibaraki	1.06	1.03	1.09
Japan	Ishikawa	1.05	1.02	1.08
Japan	Iwate	1.07	1.04	1.11
Japan	Kagawa	1.04	1.01	1.07
Japan	Kagoshima	1.03	1	1.05
Japan	Kanagawa	1.05	1.03	1.07
Japan	Kochi	1.03	1	1.06
Japan	Kumamoto	1.01	0.99	1.04
Japan	Kyoto	1.05	1.03	1.07
Japan	Mie	1.05	1.03	1.08
Japan	Miyagi	1.06	1.03	1.09
Japan	Miyazaki	1.03	1	1.06
Japan	Nagano	1.05	1.02	1.07
Japan	Nagasaki	1.02	0.99	1.04
Japan	Nara	1.04	1.01	1.06
Japan	Niigata	1.08	1.06	1.11
Japan	Oita	1.04	1.02	1.07
Japan	Okayama	1.03	1.01	1.06
Japan	Okinawa	1.01	0.98	1.05
Japan	Saga	1.02	0.99	1.05
Japan	Saitama	1.08	1.06	1.1
Japan	Shiga	1.05	1.02	1.08
Japan	Shimane	1.04	1.01	1.07
Japan	Shizuoka	1.04	1.01	1.06
Japan	Tochigi	1.07	1.04	1.1
Japan	Tokushima	1.04	1.01	1.07
Japan	Tottori	1.07	1.03	1.1
Japan	Toyama	1.05	1.02	1.08
Japan	Wakayama	1.03	1	1.06
Japan	Yamagata	1.08	1.05	1.11
Japan	Yamaguchi	1.04	1.02	1.07
Japan	Yamanashi	1.05	1.01	1.08
Moldova	Anenii Noi	1.18	1.07	1.29

Moldova	Cahul	1.21	1.1	1.33
Moldova	Chisinau	1.2	1.09	1.31
Moldova	Falesti	1.16	1.06	1.28
Philippines	Cebu	1.16	1.09	1.23
Philippines	Davao	1.15	1.08	1.22
Philippines	Manila	1.15	1.09	1.22
Philippines	Quezon	1.16	1.1	1.23
South Korea	Busan	1.02	0.99	1.06
South Korea	Daegu	1.07	1.03	1.11
South Korea	Daejeon	1.07	1.03	1.11
South Korea	Gwangju	1.08	1.04	1.12
South Korea	Incheon	1.07	1.04	1.12
South Korea	Seoul	1.08	1.05	1.12
South Korea	Ulsan	1.05	1.01	1.1
Spain	Vitoria	1.17	1.11	1.22
Spain	A Coruna	1.15	1.1	1.2
Spain	Albacete	1.21	1.16	1.26
Spain	Alicante	1.23	1.17	1.28
Spain	Almeria	1.24	1.19	1.3
Spain	Avila	1.17	1.11	1.22
Spain	Badajoz	1.23	1.18	1.29
Spain	Barcelona	1.17	1.13	1.21
Spain	Bilbao	1.18	1.13	1.23
Spain	Burgos	1.15	1.1	1.2
Spain	Caceres	1.23	1.18	1.29
Spain	Cadiz	1.25	1.19	1.3
Spain	Castellon	1.21	1.16	1.27
Spain	Ceuta	1.23	1.17	1.28
Spain	Ciudad Real	1.25	1.2	1.31
Spain	Cordoba	1.29	1.24	1.35
Spain	Cuenca	1.2	1.15	1.25
Spain	Girona	1.19	1.14	1.24
Spain	Granada	1.21	1.17	1.26
Spain	Guadalajara	1.21	1.16	1.27
Spain	Huelva	1.25	1.2	1.31
Spain	Huesca	1.22	1.17	1.27
Spain	Jaen	1.25	1.2	1.31
Spain	Leon	1.13	1.07	1.18
Spain	Lleida	1.2	1.15	1.25
Spain	Logrono	1.19	1.14	1.24
Spain	Lugo	1.17	1.11	1.23
Spain	Madrid	1.27	1.23	1.3
Spain	Malaga	1.23	1.18	1.28
Spain	Melilla	1.23	1.18	1.29
Spain	Murcia	1.17	1.12	1.22
Spain	Ourense	1.18	1.13	1.23

Spain	Oviedo	1.15	1.1	1.2
Spain	Palma Mallorca	1.2	1.15	1.24
Spain	Palmas G. Canaria	1.19	1.14	1.24
Spain	Pamplona	1.17	1.13	1.22
Spain	Pontevedra	1.17	1.12	1.23
Spain	Salamanca	1.18	1.13	1.23
Spain	San Sebastian	1.17	1.12	1.23
Spain	Santander	1.15	1.11	1.21
Spain	Segovia	1.2	1.14	1.25
Spain	Sevilla	1.3	1.25	1.35
Spain	Soria	1.16	1.11	1.22
Spain	Tarragona	1.22	1.17	1.27
Spain	Tenerife	1.27	1.22	1.33
Spain	Teruel	1.18	1.13	1.23
Spain	Toledo	1.24	1.19	1.3
Spain	Valencia	1.19	1.14	1.23
Spain	Valladolid	1.22	1.17	1.27
Spain	Zamora	1.19	1.14	1.25
Spain	Zaragoza	1.23	1.18	1.28
Sweden	Stockholm	1.01	0.94	1.08
Taiwan	Kaohsiung	1.05	1	1.1
Taiwan	Taichung	1.05	1	1.1
Taiwan	Taipei	1.07	1.03	1.11
Thailand	Amnat Charoen	1.04	0.99	1.09
Thailand	Ayutthaya	1.06	1.01	1.11
Thailand	Bangkok	1.08	1.03	1.12
Thailand	Buri Ram	1.06	1.01	1.1
Thailand	Chachoengsao	1.04	0.99	1.09
Thailand	Chaiyaphum	1.07	1.02	1.11
Thailand	Chanthaburi	1.05	1	1.1
Thailand	Chiang Mai	1.04	1	1.09
Thailand	Chiang Rai	1.04	0.98	1.1
Thailand	Chon Buri	1.06	1.01	1.1
Thailand	Chumphon	1.07	1.02	1.12
Thailand	Kalasin	1.06	1.02	1.11
Thailand	Kamphaeng Phet	1.06	1.01	1.11
Thailand	Kanchanaburi	1.07	1.02	1.12
Thailand	Khon Kaen	1.04	1	1.09
Thailand	Krabi	1.06	1.01	1.11
Thailand	Lampang	1.09	1.05	1.14
Thailand	Lamphun	1.05	1.01	1.1
Thailand	Lop Buri	1.04	0.99	1.09
Thailand	Maha Sarakham	1.09	1.04	1.14
Thailand	Mukdahan	1.05	1	1.1
Thailand	Nakhon Pathom	1.07	1.02	1.11
Thailand	Nakhon Phanom	1.05	1	1.09

Thailand	Nakhon Ratchasima	1.07	1.03	1.11
Thailand	Nakhon Sawan	1.08	1.03	1.13
Thailand	Nakhon Si Thammarat	1.05	1	1.11
Thailand	Nan	1.06	1.02	1.11
Thailand	Narathiwat	1.05	1	1.1
Thailand	Nong Bua Lam Phu	1.06	1.01	1.11
Thailand	Nong Khai	1.05	1	1.1
Thailand	Nonthaburi	1.08	1.03	1.12
Thailand	Pathum Thani	1.07	1.03	1.12
Thailand	Pattani	1.05	1.01	1.1
Thailand	Phayao	1.05	1.01	1.09
Thailand	Phetchabun	1.04	0.98	1.09
Thailand	Phetchaburi	1.05	1	1.1
Thailand	Phichit	1.07	1.02	1.11
Thailand	Phitsanulok	1.07	1.03	1.12
Thailand	Phrae	1.05	1.01	1.1
Thailand	Prachin Buri	1.07	1.02	1.12
Thailand	Prachuap Khiri Khan	1.06	1.01	1.1
Thailand	Ratchaburi	1.06	1.02	1.11
Thailand	Rayong	1.04	0.99	1.1
Thailand	Roi Et	1.06	1.02	1.11
Thailand	Sa Kaeo	1.06	1.01	1.1
Thailand	Sakon Nakhon	1.05	1.01	1.1
Thailand	Samut Sakhon	1.06	1.01	1.1
Thailand	Samutprakan	1.08	1.03	1.12
Thailand	Saraburi	1.06	1.02	1.11
Thailand	Si Sa Ket	1.05	1	1.09
Thailand	Songkhla	1.06	1.02	1.11
Thailand	Sukhothai	1.08	1.03	1.13
Thailand	Suphanburi	1.08	1.03	1.13
Thailand	Surat Thani	1.05	1.01	1.1
Thailand	Surin	1.06	1.01	1.1
Thailand	Tak	1.06	1.02	1.11
Thailand	Trang	1.06	1.02	1.11
Thailand	Ubon Ratchathani	1.08	1.03	1.12
Thailand	Udon Thani	1.07	1.03	1.12
Thailand	Uttaradit	1.07	1.02	1.11
Thailand	Yala	1.05	1	1.11
Thailand	Yasothon	1.06	1.01	1.11
UK	East	1.04	1.02	1.07
UK	East Midlands	1.02	1	1.04
UK	London	1.09	1.04	1.13
UK	North East	1	0.97	1.03
UK	North West	1.02	0.99	1.05
UK	South East	1.03	1	1.05
UK	South West	1	0.98	1.02

UK	Wales	1.01	0.99	1.04
UK	West Midlands	1.01	0.99	1.03
UK	Yorkshire & Humber	1.03	1	1.05
USA	Akron, OH	1.02	0.98	1.06
USA	Albuquerque, NM	1.02	0.98	1.06
USA	Allentown-Bethlehem, PA	1.03	0.99	1.08
USA	Atlanta, GA	1.05	1.02	1.07
USA	Atlantic City, NJ	1.03	0.99	1.07
USA	Austin, TX	1.03	0.99	1.07
USA	Bakersfield, CA	1.04	1	1.08
USA	Baltimore, MD	1.06	1.03	1.09
USA	Barnstable-Yarmouth, MA	1.04	1	1.08
USA	Baton Rouge, LA	1.04	1	1.08
USA	Bergen-Passaic, NJ	1.06	1.03	1.1
USA	Birmingham, AL	1.04	1.01	1.07
USA	Boston, MA	1.03	1	1.06
USA	Brownsville, TX	1.02	0.98	1.06
USA	Buffalo, NY	1.05	1.02	1.09
USA	Canton-Massillon, OH	1.03	0.99	1.07
USA	Charleston, WV	1.04	1	1.08
USA	Charlotte, NC	0.98	0.95	1.02
USA	Chattanooga, TN	1.02	0.98	1.06
USA	Chicago, IL	1.04	1.01	1.07
USA	Cincinnati, OH	1.05	1.02	1.08
USA	Cleveland, OH	1.04	1.01	1.07
USA	Columbia, SC	1.03	1	1.07
USA	Columbus, OH	1.02	0.99	1.06
USA	Dallas, TX	1.03	1	1.06
USA	Dayton, OH	1.03	0.99	1.07
USA	Daytona Beach, FL	1.01	0.98	1.05
USA	Denver, CO	1.05	1.01	1.09
USA	Des Moines, IA	1.03	0.99	1.07
USA	Detroit, MI	1.07	1.04	1.09
USA	Dutchess County, NY	1.04	1	1.08
USA	El Paso, TX	1.03	0.99	1.07
USA	Erie, PA	1.06	1.02	1.1
USA	Flint, MI	1.02	0.98	1.06
USA	Fort Myers-Cape Coral, FL	1.03	0.99	1.07
USA	Fort Pierce-Port St. Lucie, FL	1.02	0.99	1.06
USA	Fort Worth-Arlington, TX	1.04	1	1.07
USA	Fresno, CA	1.04	1	1.08
USA	Ft. Lauderdale, FL	1	0.96	1.04
USA	Galveston, TX	1.02	0.98	1.06
USA	Gary, IN	1.02	0.98	1.06
USA	Grand Rapids, MI	1.05	1.01	1.09
USA	Greensboro, NC	1.01	0.98	1.05

USA	Greenville, SC	1.04	1	1.08
USA	Hamilton, OH	1.03	0.99	1.07
USA	Harrisburg-Carlisle, PA	1.03	0.99	1.07
USA	Hartford, CT	1.05	1.02	1.09
USA	Honolulu, HI	1.01	0.96	1.05
USA	Houston, TX	1	0.98	1.03
USA	Indianapolis, IN	1.04	1.01	1.08
USA	Jacksonville, FL	1	0.97	1.04
USA	Jersey City, NJ	1.04	1	1.07
USA	Kansas City, MO-KS	1.07	1.04	1.1
USA	Knoxville, TN	1.04	1	1.08
USA	Lakeland-Winter Haven, FL	1.05	1.01	1.09
USA	Lancaster, PA	1.03	1	1.07
USA	Lansing, MI	1.05	1.01	1.1
USA	Las Vegas, NV-AZ	1.01	0.97	1.04
USA	Little Rock, AR	1.04	1.01	1.08
USA	Los Angeles, CA	1.05	1.03	1.08
USA	Louisville, KY	1.04	1	1.07
USA	Lubbock, TX	1.01	0.97	1.06
USA	Madison, WI	1.03	0.99	1.08
USA	McAllen-Edinburg-Mission, TX	1	0.96	1.04
USA	Melbourne-Titusville-Palm Bay, FL	1.03	1	1.07
USA	Memphis, TN	1.01	0.98	1.05
USA	Miami, FL	1.04	1	1.07
USA	Middlesex, NJ	1.04	1	1.08
USA	Milwaukee, WI	1.04	1	1.07
USA	Minneapolis-St. Paul, MN	1.02	0.99	1.05
USA	Mobile, AL	1.03	0.99	1.07
USA	Monmouth-Ocean, NJ	1.02	0.98	1.05
USA	Myrtle Beach, SC	1.03	0.99	1.07
USA	Naples, FL	1.02	0.97	1.06
USA	Nashua, NH	1.03	0.99	1.07
USA	Nashville, TN	1.02	0.99	1.06
USA	Nassau-Suffolk, NY	1.02	1	1.05
USA	New Haven-Meriden, CT	1.08	1.05	1.12
USA	New London, CT	1.06	1.02	1.11
USA	New York, NY	1.12	1.09	1.15
USA	Newark, NJ	1.07	1.03	1.1
USA	Newburgh, NY	1.04	1	1.08
USA	Oakland, CA	1.06	1.03	1.1
USA	Ocala, FL	1.02	0.98	1.06
USA	Oklahoma City, OK	1.01	0.98	1.05
USA	Omaha, NE	1.03	0.99	1.07
USA	Orange County, CA	1.03	1	1.06
USA	Orlando, FL	1.01	0.97	1.04
USA	Pensacola, FL	1.02	0.98	1.06

USA	Philadelphia, PA-NJ	1.05	1.03	1.08
USA	Phoenix, AZ	1.04	1	1.07
USA	Pittsburgh, PA	1.06	1.03	1.09
USA	Portland, ME	1.07	1.02	1.11
USA	Portland, OR	1.07	1.04	1.1
USA	Providence-Fall River, RI-MA	1.04	1	1.09
USA	Punta Gorda, FL	1.03	0.99	1.07
USA	Raleigh, NC	1.02	0.98	1.05
USA	Reading, PA	1.04	1	1.08
USA	Riverside-San Bernardino, CA	1.03	1	1.05
USA	Rochester, NY	1.05	1.02	1.09
USA	Rockford, IL	1.03	0.99	1.07
USA	Sacramento, CA	1.05	1.01	1.08
USA	Saginaw, MI	1.05	1	1.09
USA	Salinas, CA	1.04	1	1.09
USA	Salt Lake City, UT	1.03	1	1.07
USA	San Antonio, TX	1.02	0.99	1.05
USA	San Diego, CA	1.03	1	1.06
USA	San Francisco, CA	1.04	1.01	1.08
USA	San Jose, CA	1.06	1.03	1.1
USA	Sarasota-Bradenton, FL	0.99	0.96	1.03
USA	Scranton--Wilkes-Barre--Hazleton, PA	1.04	1	1.07
USA	Seattle, WA	1.08	1.05	1.12
USA	Shreveport, LA	1.02	0.98	1.06
USA	Spokane, WA	1.06	1.02	1.1
USA	Springfield, MA	1.05	1.01	1.09
USA	St. Louis, MO-IL	1.03	1	1.05
USA	Stamford-Norwalk, CT	1.05	1.02	1.09
USA	Stockton-Lodi, CA	1.02	0.99	1.06
USA	Syracuse, NY	1.04	1	1.08
USA	Tacoma, WA	1.04	1	1.08
USA	Tampa-St. Petersburg-Clearwater, FL	1.02	0.99	1.05
USA	Toledo, OH	1.04	1	1.07
USA	Trenton, NJ	1.03	0.99	1.07
USA	Tucson, AZ	1.02	0.98	1.06
USA	Tulsa, OK	1	0.97	1.04
USA	Utica-Rome, NY	1.02	0.98	1.07
USA	Ventura County, CA	1.05	1.01	1.09
USA	Virginia Beach, VA	1.04	1.01	1.07
USA	Washington, DC-MD-VA	1.03	0.99	1.06
USA	West Palm Beach-Boca Raton, FL	1.02	0.98	1.05
USA	Wichita, KS	1.03	1	1.07
USA	Wilmington, DE	1.03	0.99	1.07
USA	Worcester, MA	1.05	1.02	1.09
USA	York, PA	1.04	1	1.08
USA	Youngstown-Warren, OH	1.05	1.01	1.09

Vietnam	Ho Chi Minh City	1.13	1.03	1.25
Vietnam	Hue	1.12	1.02	1.24

Table C: Mean percent change (and 95% eCI) of heatwave-related excess deaths between period 2031-2080 and period 1971-2020, in 20 countries/regions under RCP2.6, RCP4.5, RCP6.0 and RCP8.5 scenarios and three population scenarios (low variant, median variant, and high variant), with assumption of non-adaptation.

**Country/region**	**% Percent change (95% eCI)**					
	**RCP2.6**			**RCP4.5**		
	**Low variant population**	**Median variant population**	**High variant population**	**Low variant population**	**Median variant population**	**High variant population**
Australia	176 (123, 228)	213 (154, 272)	255 (188, 322)	216 (124, 308)	259 (154, 363)	307 (188, 425)
Brazil	255 (-3, 512)	313 (13, 612)	380 (32, 728)	370 (107, 632)	447 (141, 752)	536 (181, 892)
Canada	125 (63, 186)	155 (85, 225)	188 (109, 268)	206 (147, 266)	247 (180, 315)	293 (217, 370)
Chile	123 (37, 209)	157 (58, 256)	197 (83, 311)	170 (74, 266)	211 (100, 322)	259 (131, 386)
Mainland, China	84 (60, 108)	111 (83, 139)	141 (110, 173)	128 (99, 158)	161 (127, 195)	199 (160, 237)
Colombia	452 (225, 678)	545 (280, 810)	652 (343, 960)	814 (189, 1440)	968 (237, 1699)	1146 (293, 1998)
Finland	67 (-59, 193)	90 (-54, 233)	116 (-47, 279)	136 (13, 259)	169 (29, 309)	205 (46, 364)
Ireland	141 (115, 167)	175 (145, 204)	212 (179, 246)	201 (176, 226)	243 (214, 272)	290 (257, 322)
Italy	45 (31, 60)	64 (48, 80)	85 (67, 103)	86 (67, 106)	110 (89, 132)	137 (113, 162)
Japan	33 (6, 59)	50 (20, 79)	69 (35, 102)	60 (46, 75)	81 (65, 98)	104 (86, 123)
Moldova	5 (-5, 16)	23 (11, 36)	44 (30, 59)	35 (31, 38)	58 (53, 62)	84 (79, 89)
Philippines	349 (87, 611)	427 (120, 734)	515 (156, 874)	680 (371, 988)	815 (453, 1177)	969 (546, 1392)
South Korea	93 (65, 121)	119 (87, 150)	147 (112, 183)	111 (89, 133)	139 (114, 164)	171 (143, 199)
Spain	46 (24, 68)	65 (40, 90)	86 (59, 114)	95 (56, 133)	120 (77, 164)	149 (100, 198)
Sweden	99 (-48, 246)	126 (-41, 294)	157 (-33, 347)	199 (65, 334)	241 (88, 394)	287 (113, 461)
Taiwan	111 (98, 124)	141 (126, 156)	174 (157, 191)	187 (143, 231)	227 (177, 278)	272 (215, 330)
Thailand	88 (5, 172)	117 (21, 214)	151 (39, 262)	158 (58, 259)	198 (82, 314)	244 (110, 378)
UK	96 (78, 113)	123 (103, 143)	154 (131, 176)	131 (104, 158)	164 (133, 194)	200 (165, 235)
USA	129 (73, 185)	162 (98, 226)	200 (127, 273)	212 (87, 336)	257 (115, 400)	309 (145, 472)
Vietnam	210 (184, 235)	258 (228, 287)	312 (278, 346)	330 (245, 416)	398 (298, 497)	473 (359, 587)
	**RCP6.0**			**RCP8.5**		

**Country/region**	**Low variant population**	**Median variant population**	**High variant population**	**Low variant population**	**Median variant population**	**High variant population**
Australia	247 (107, 387)	294 (135, 453)	346 (166, 526)	343 (191, 495)	404 (231, 577)	471 (275, 667)
Brazil	443 (-85, 971)	533 (-82, 1147)	636 (-79, 1351)	605 (48, 1161)	720 (73, 1367)	854 (101, 1607)
Canada	212 (153, 271)	254 (187, 321)	301 (225, 377)	332 (263, 401)	390 (312, 468)	455 (366, 543)
Chile	183 (61, 304)	226 (86, 366)	276 (114, 437)	323 (150, 496)	388 (189, 588)	462 (232, 692)
Mainland, China	144 (110, 179)	180 (140, 220)	220 (175, 266)	212 (140, 284)	257 (174, 339)	308 (214, 402)
Colombia	912 (486, 1338)	1083 (585, 1581)	1279 (699, 1859)	1455 (431, 2479)	1717 (520, 2914)	2019 (623, 3414)
Finland	118 (9, 227)	148 (24, 272)	182 (41, 323)	165 (2, 327)	201 (17, 386)	242 (33, 452)
Ireland	241 (224, 258)	289 (269, 308)	342 (320, 363)	333 (278, 388)	394 (331, 456)	461 (390, 532)
Italy	111 (91, 132)	139 (116, 162)	169 (143, 195)	173 (147, 199)	208 (179, 238)	248 (214, 281)
Japan	78 (56, 100)	101 (76, 126)	127 (99, 154)	115 (73, 156)	143 (96, 189)	174 (121, 227)
Moldova	41 (32, 51)	65 (54, 77)	93 (80, 106)	80 (71, 88)	110 (100, 120)	146 (134, 158)
Philippines	604 (42, 1165)	726 (67, 1385)	865 (95, 1635)	928 (131, 1724)	1106 (171, 2042)	1309 (217, 2401)
South Korea	134 (93, 175)	165 (119, 211)	200 (148, 252)	192 (158, 225)	231 (193, 268)	274 (231, 317)
Spain	130 (88, 172)	160 (112, 208)	194 (140, 247)	207 (166, 248)	247 (200, 294)	292 (239, 344)
Sweden	186 (51, 321)	226 (72, 379)	270 (96, 444)	227 (61, 392)	272 (84, 460)	322 (108, 536)
Taiwan	194 (168, 220)	235 (205, 265)	281 (247, 315)	330 (244, 416)	390 (292, 488)	457 (345, 569)
Thailand	147 (21, 274)	185 (39, 331)	229 (61, 398)	266 (89, 443)	322 (118, 527)	387 (151, 624)
UK	174 (149, 199)	212 (184, 241)	256 (224, 288)	259 (231, 286)	309 (278, 340)	365 (330, 401)
USA	231 (110, 351)	279 (141, 417)	333 (175, 491)	355 (207, 503)	422 (162, 682)	496 (302, 690)
Vietnam	325 (266, 383)	391 (323, 458)	465 (387, 543)	531 (502, 561)	630 (249, 1011)	741 (701, 780)

Table D: Mean percent change (and 95% eCI) of heatwave-related excess deaths between period 2031-2080 and period 1971-2020, in 20 countries/regions under RCP2.6, RCP4.5, RCP6.0 and RCP8.5 scenarios and three population scenarios (low variant, median variant, and high variant), with assumption of full adaptation.

Country/region	% Percent change (95% eCI)					
	RCP2.6			RCP4.5		
	Low variant population	Median variant population	High variant population	Low variant population	Median variant population	High variant population
Australia	88 (78, 98)	114 (102, 125)	142 (129, 155)	76 (74, 79)	100 (97, 104)	127 (124, 131)
Brazil	35 (11, 58)	57 (29, 84)	82 (51, 114)	34 (13, 54)	56 (32, 80)	81 (53, 109)
Canada	45 (40, 50)	64 (59, 70)	86 (80, 92)	46 (41, 50)	65 (60, 70)	87 (81, 93)
Chile	37 (15, 60)	58 (33, 84)	83 (53, 112)	31 (17, 46)	51 (35, 68)	74 (55, 94)
Mainland, China	3 (-1, 6)	17 (13, 22)	34 (29, 39)	1 (-2, 5)	16 (12, 20)	33 (28, 37)
Colombia	23 (8, 38)	44 (27, 61)	68 (48, 87)	50 (17, 84)	75 (37, 114)	105 (59, 150)
Finland	6 (-1, 14)	21 (13, 29)	38 (28, 47)	7 (-6, 19)	21 (7, 35)	38 (22, 54)
Ireland	38 (33, 42)	57 (52, 62)	79 (73, 84)	45 (39, 50)	65 (58, 71)	87 (80, 95)
Italy	-16 (-19, -13)	-5 (-9, -2)	7 (3, 11)	-16 (-18, -13)	-5 (-8, -2)	7 (4, 11)
Japan	-22 (-25, -19)	-12 (-15, -9)	-1 (-4, 3)	-23 (-25, -21)	-13 (-16, -11)	-2 (-5, 1)
Moldova	-35 (-38, -32)	-24 (-27, -21)	-11 (-15, -8)	-34 (-36, -32)	-23 (-25, -21)	-10 (-13, -7)
Philippines	105 (89, 120)	140 (122, 158)	180 (159, 202)	104 (94, 115)	140 (127, 152)	180 (166, 195)
South Korea	2 (-1, 5)	15 (12, 19)	30 (27, 34)	0 (-2, 2)	14 (12, 16)	29 (26, 31)
Spain	-6 (-8, -3)	7 (4, 10)	20 (17, 24)	-4 (-7, -1)	9 (5, 12)	23 (19, 27)
Sweden	20 (10, 31)	37 (25, 49)	55 (42, 69)	22 (10, 34)	38 (25, 52)	57 (42, 73)
Taiwan	-2 (-6, 2)	12 (7, 16)	27 (22, 32)	-2 (-3, 0)	12 (11, 14)	27 (26, 29)
Thailand	2 (-5, 8)	17 (10, 25)	35 (27, 44)	1 (-4, 6)	17 (11, 22)	35 (28, 41)
UK	15 (11, 20)	32 (27, 36)	50 (44, 55)	15 (13, 18)	31 (28, 34)	50 (46, 53)
USA	36 (30, 41)	56 (49, 62)	78 (71, 85)	35 (30, 40)	55 (49, 61)	77 (70, 84)
Vietnam	39 (28, 51)	61 (48, 74)	86 (70, 101)	49 (23, 75)	73 (42, 103)	99 (64, 134)
	RCP6.0			RCP8.5		

Country/region	Low variant population	Median variant population	High variant population	Low variant population	Median variant population	High variant population
Australia	78 (55, 100)	102 (77, 127)	129 (100, 158)	74 (67, 82)	98 (89, 107)	125 (114, 135)
Brazil	38 (13, 62)	60 (32, 88)	86 (53, 119)	29 (2, 55)	50 (19, 81)	75 (39, 111)
Canada	47 (40, 53)	66 (59, 73)	88 (80, 96)	49 (44, 55)	69 (63, 76)	92 (85, 99)
Chile	33 (21, 45)	53 (40, 67)	77 (61, 93)	33 (11, 55)	53 (28, 78)	77 (48, 105)
Mainland, China	2 (-1, 5)	17 (13, 20)	33 (29, 38)	3 (-3, 9)	18 (11, 24)	35 (27, 42)
Colombia	48 (2, 94)	73 (19, 127)	102 (38, 165)	50 (-5, 106)	76 (11, 141)	105 (29, 180)
Finland	8 (-4, 20)	23 (9, 36)	40 (24, 55)	6 (-5, 17)	21 (9, 34)	38 (23, 52)
Ireland	41 (37, 45)	61 (57, 65)	83 (78, 88)	40 (34, 46)	60 (53, 67)	82 (74, 90)
Italy	-16 (-18, -13)	-5 (-8, -2)	7 (4, 10)	-16 (-19, -13)	-5 (-8, -1)	7 (3, 11)
Japan	-22 (-24, -20)	-12 (-14, -10)	-1 (-3, 2)	-21 (-24, -19)	-11 (-14, -8)	0 (-3, 4)
Moldova	-34 (-35, -32)	-22 (-24, -20)	-9 (-11, -7)	-34 (-35, -33)	-23 (-24, -22)	-10 (-11, -9)
Philippines	111 (96, 126)	148 (130, 166)	190 (169, 211)	101 (88, 113)	136 (121, 150)	175 (158, 192)
South Korea	1 (-1, 3)	14 (12, 16)	29 (27, 31)	2 (-2, 6)	16 (11, 21)	31 (26, 36)
Spain	-4 (-7, -2)	8 (5, 11)	22 (18, 25)	-4 (-8, 0)	9 (4, 13)	23 (18, 27)
Sweden	23 (15, 31)	40 (31, 49)	59 (49, 69)	21 (15, 28)	38 (31, 45)	57 (49, 65)
Taiwan	0 (-3, 3)	14 (11, 17)	30 (26, 33)	0 (-2, 1)	14 (12, 16)	30 (28, 32)
Thailand	2 (-4, 8)	17 (10, 24)	35 (27, 44)	0 (-10, 10)	15 (4, 27)	33 (20, 47)
UK	17 (14, 20)	33 (30, 37)	52 (48, 56)	15 (12, 18)	31 (28, 34)	49 (46, 53)
USA	36 (31, 41)	56 (50, 61)	78 (71, 84)	37 (30, 44)	57 (49, 65)	79 (71, 88)
Vietnam	44 (38, 51)	67 (59, 75)	92 (83, 101)	43 (39, 48)	66 (60, 71)	91 (84, 97)

Table E: Annual death number (and 95% eCI) in relation to heatwaves during 1971-2020 in 20 countries/regions.

Country/region		95% eCI	
	Mean	Low	Up
Australia	142	112	173
Brazil	732	570	893
Canada	300	272	329
Chile	70	59	82
China	1244	1149	1338
Colombia	21	4	38
Finland	40	37	43
Ireland	12	8	16
Italy	799	740	858
Japan	2034	1890	2178
Moldova	48	42	55
Philippines	322	221	423
South Korea	258	232	285
Spain	1413	1303	1523
Sweden	6	5	6
Taiwan	136	124	148
Thailand	473	410	536
UK	540	489	590
USA	1756	1576	1935
Vietnam	160	142	177

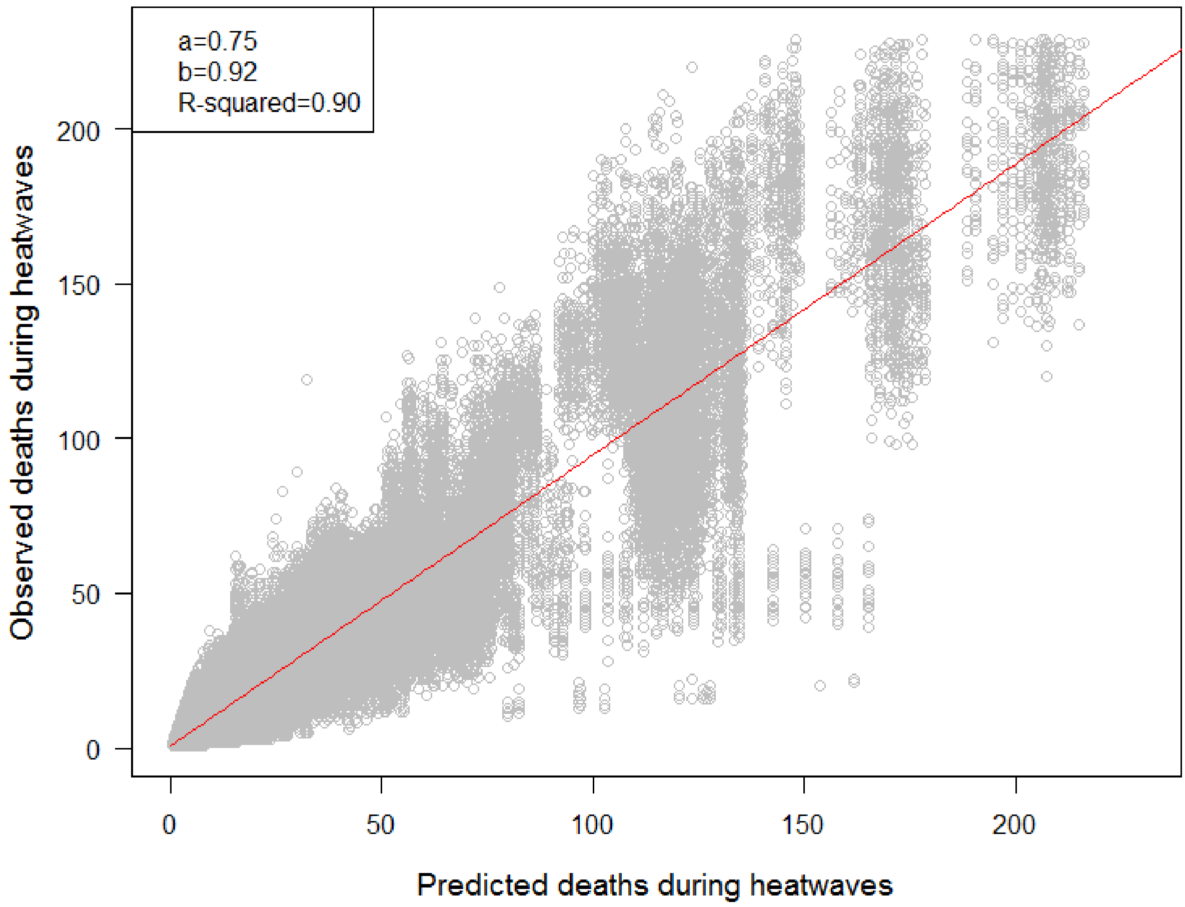

Fig A: Scatterplots of cross-validation of modelling fitting for daily deaths during heatwaves.

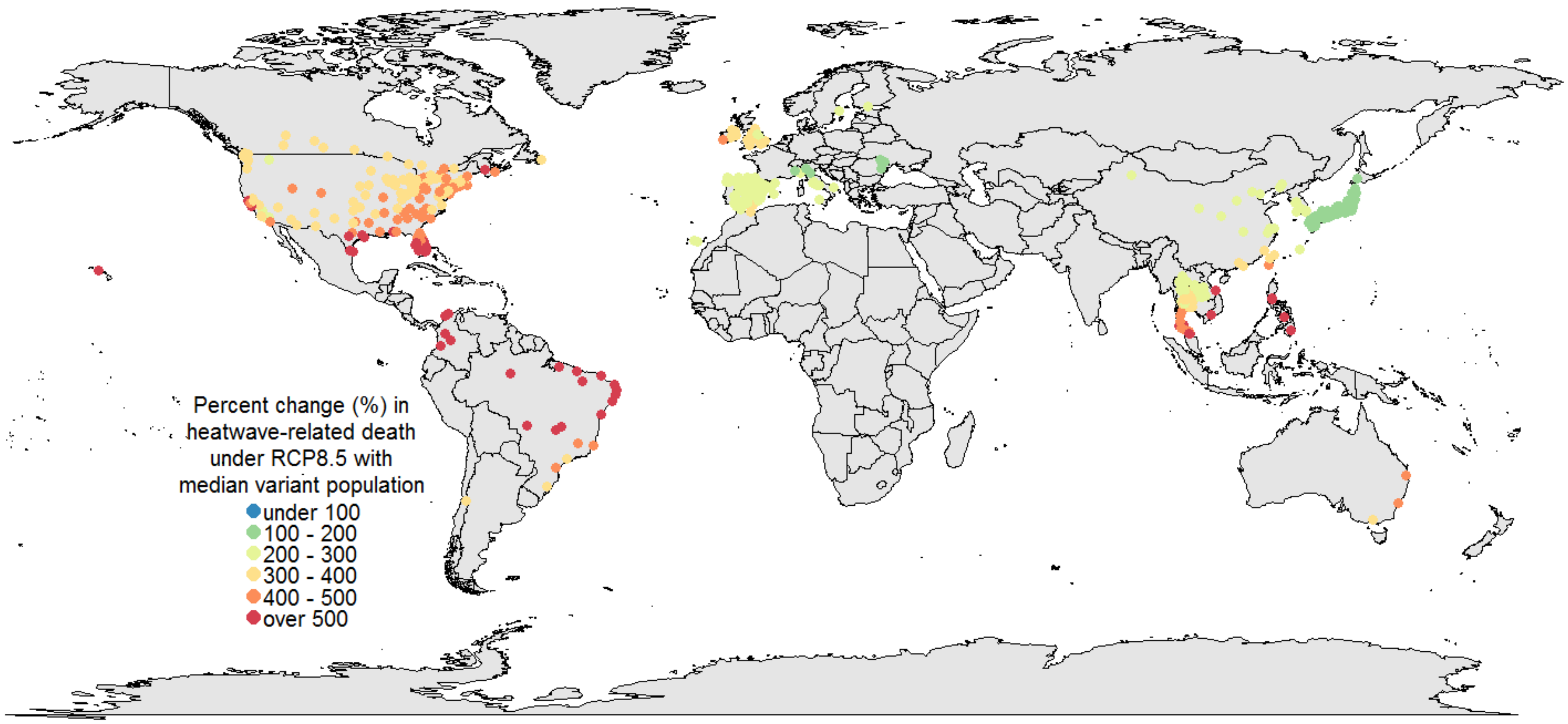

Fig B: Percent change of heatwave-related excess deaths in period 2031-2080 in comparison to period 1971-2020 under RCP8.5 scenario and median variant population scenario, in 412 communities, without any adaptation to future climate change.

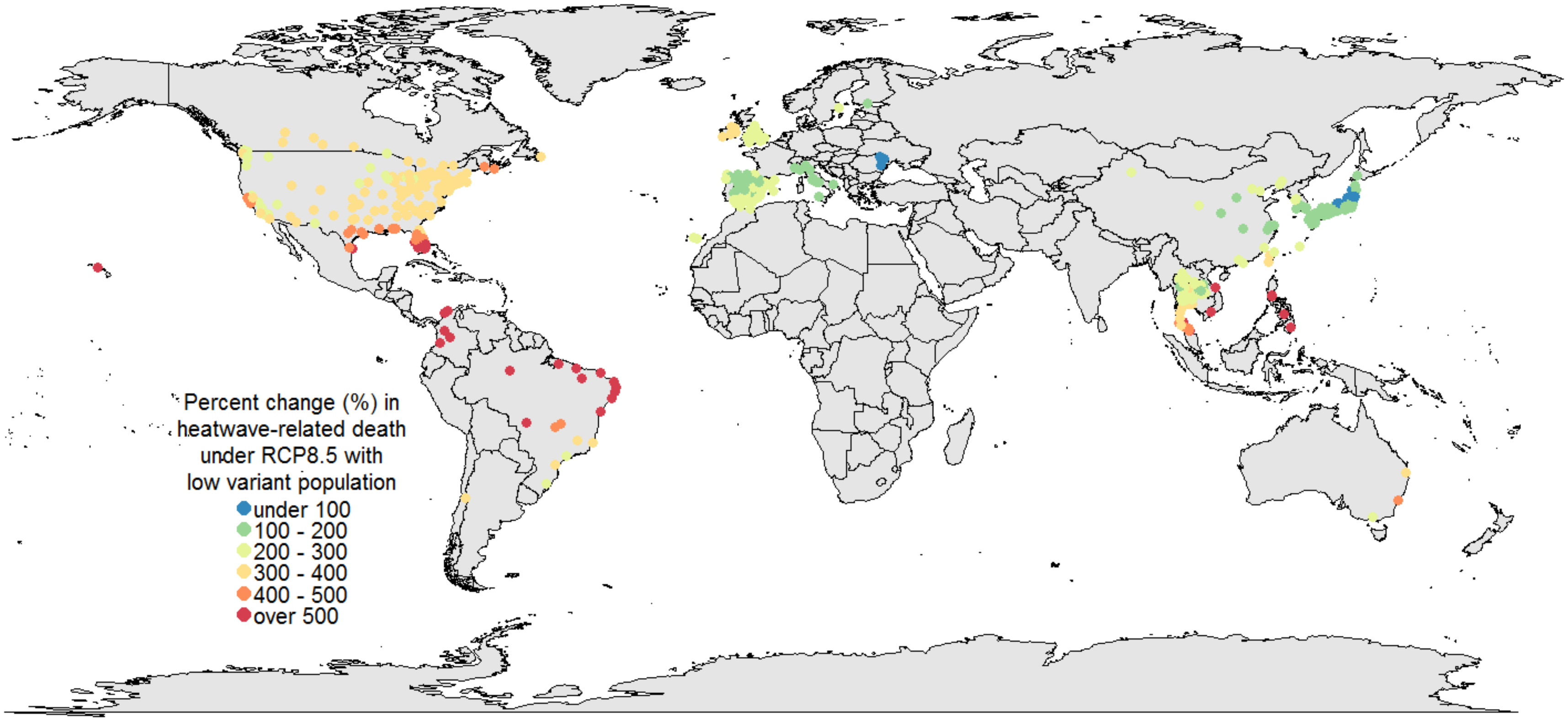

Fig C: Percent change of heatwave-related excess deaths in period 2031-2080 in comparison to period 1971-2020 under RCP8.5 scenario and low variant population scenario, in 412 communities, without any adaptation to future climate change.

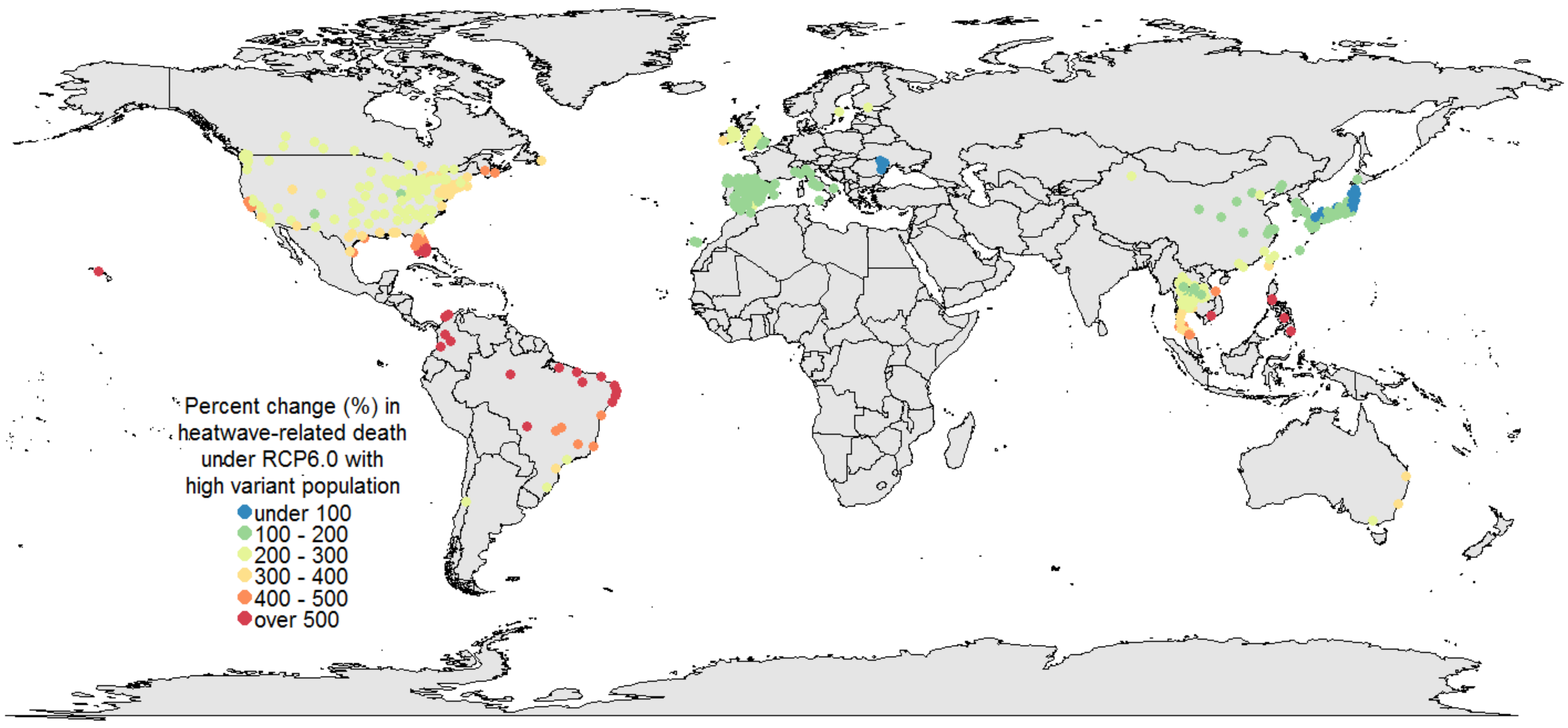

Fig D: Percent change of heatwave-related excess deaths in period 2031-2080 in comparison to period 1971-2020 under RCP6.0 scenario and high variant population scenario, in 412 communities, without any adaptation to future climate change.

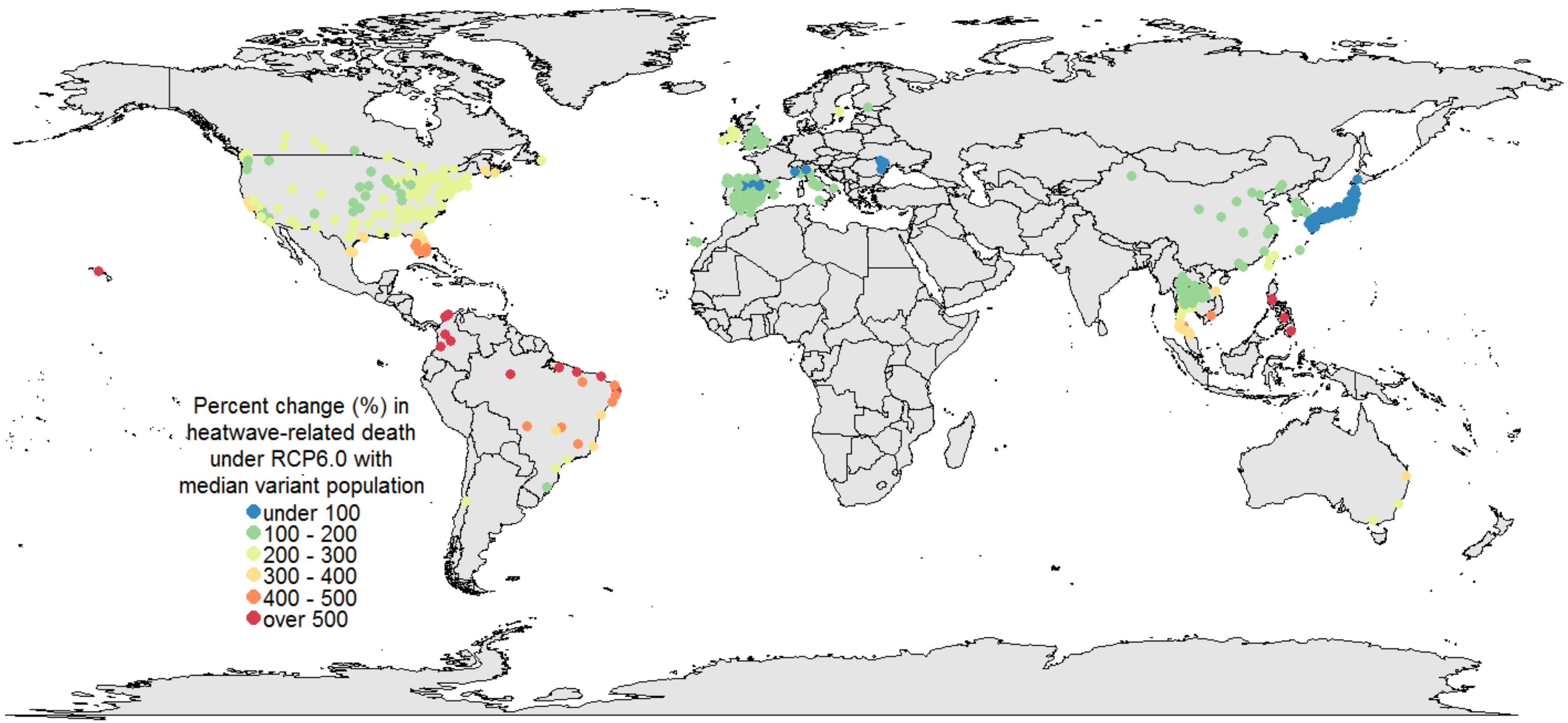

Fig E: Percent change of heatwave-related excess deaths in period 2031-2080 in comparison to period 1971-2020 under RCP6.0 scenario and median variant population scenario, in 412 communities, without any adaptation to future climate change.

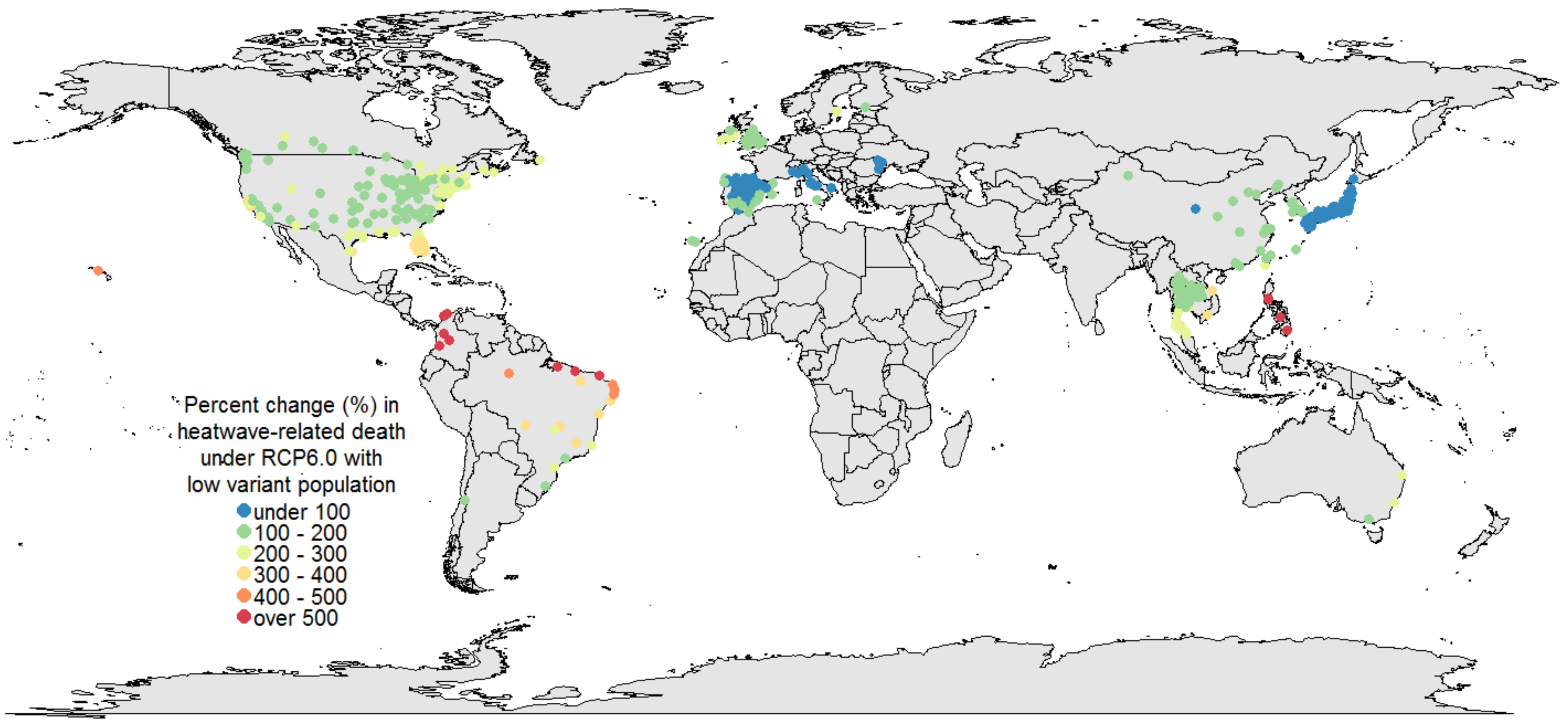

Fig F: Percent change of heatwave-related excess deaths in period 2031-2080 in comparison to period 1971-2020 under RCP6.0 scenario and low variant population scenario, in 412 communities, without any adaptation to future climate change.

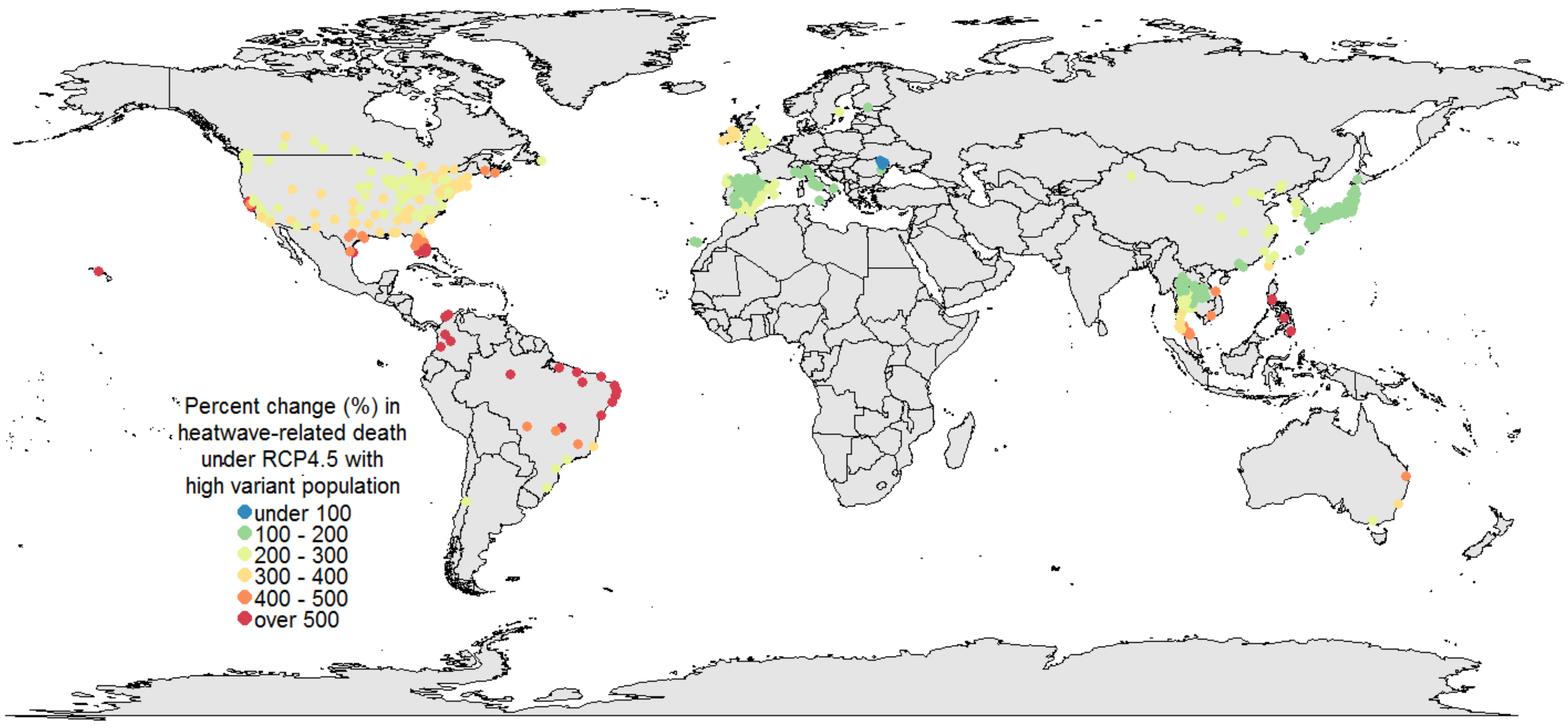

Fig G: Percent change of heatwave-related excess deaths in period 2031-2080 in comparison to period 1971-2020 under RCP4.5 scenario and high variant population scenario, in 412 communities, without any adaptation to future climate change.

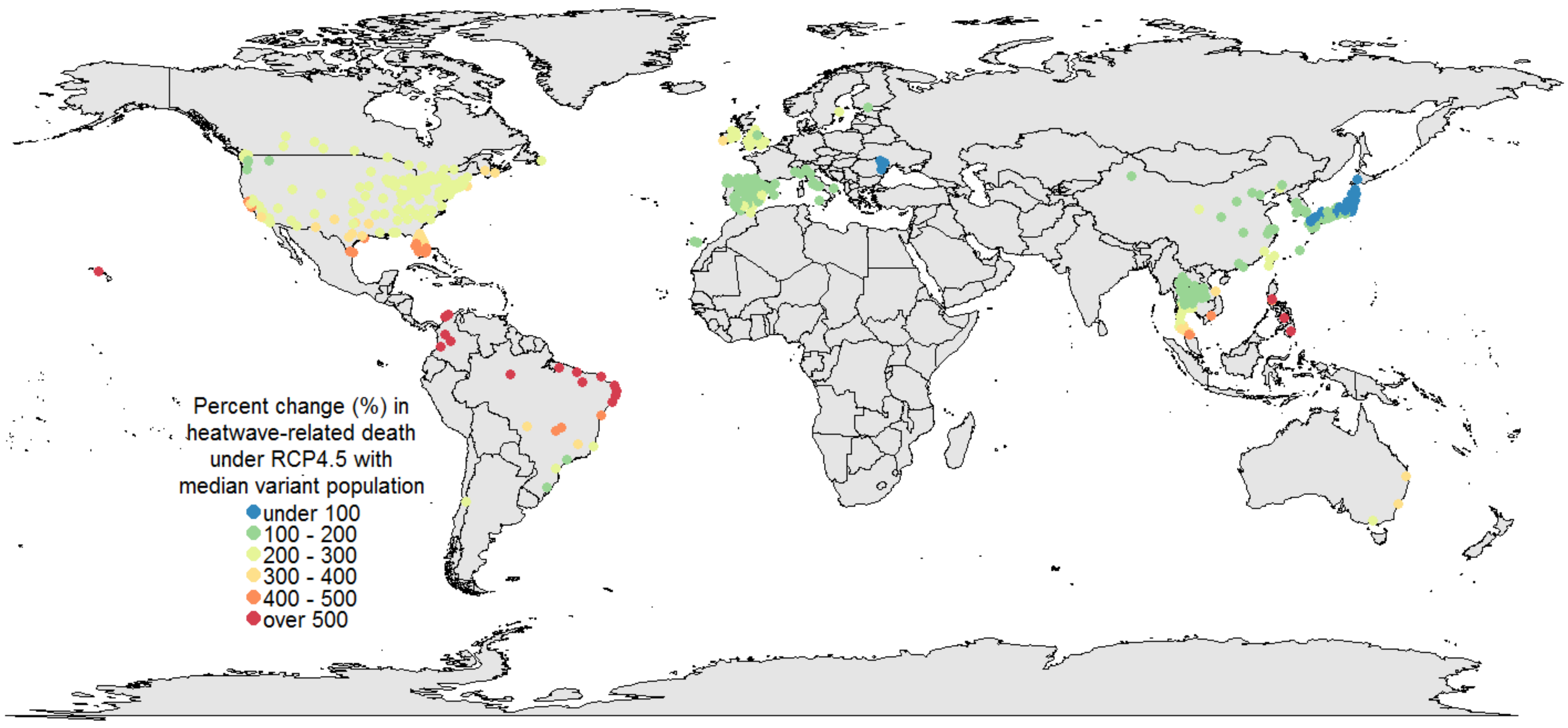

Fig H: Percent change of heatwave-related excess deaths in period 2031-2080 in comparison to period 1971-2020 under RCP4.5 scenario and median variant population scenario, in 412 communities, without any adaptation to future climate change.

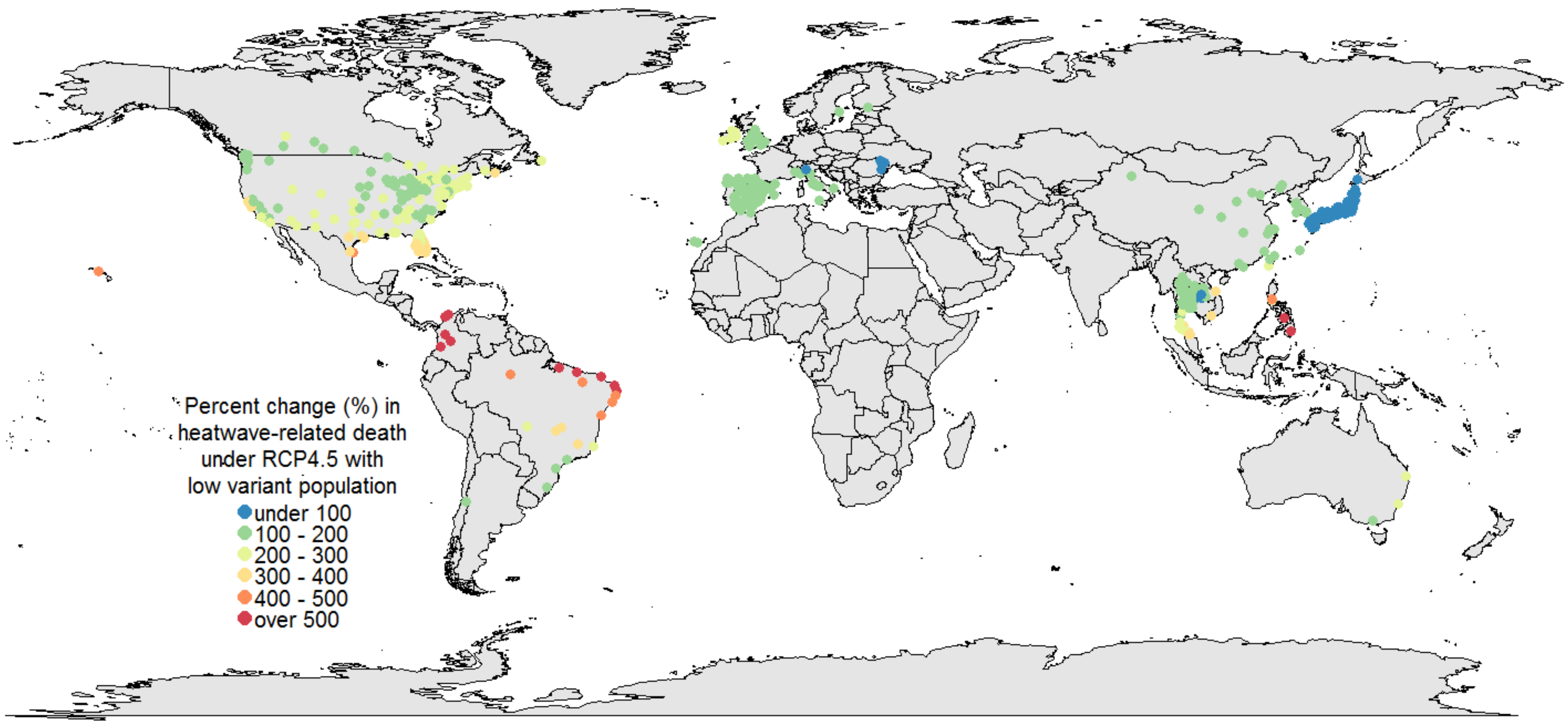

Fig I: Percent change of heatwave-related excess deaths in period 2031-2080 in comparison to period 1971-2020 under RCP4.5 scenario and low variant population scenario, in 412 communities, without any adaptation to future climate change.

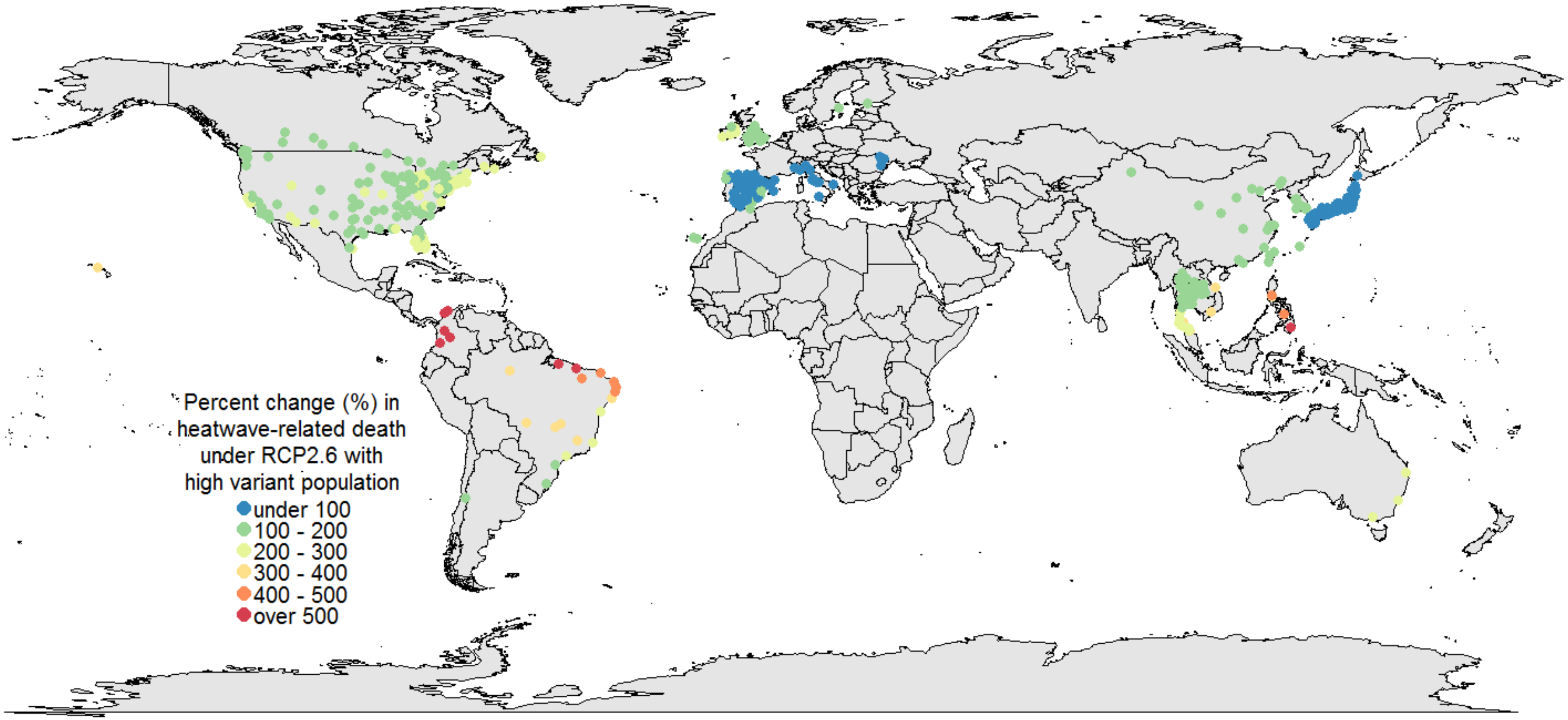

Fig J: Percent change of heatwave-related excess deaths in period 2031-2080 in comparison to period 1971-2020 under RCP2.6 scenario and high variant population scenario, in 412 communities, without any adaptation to future climate change.

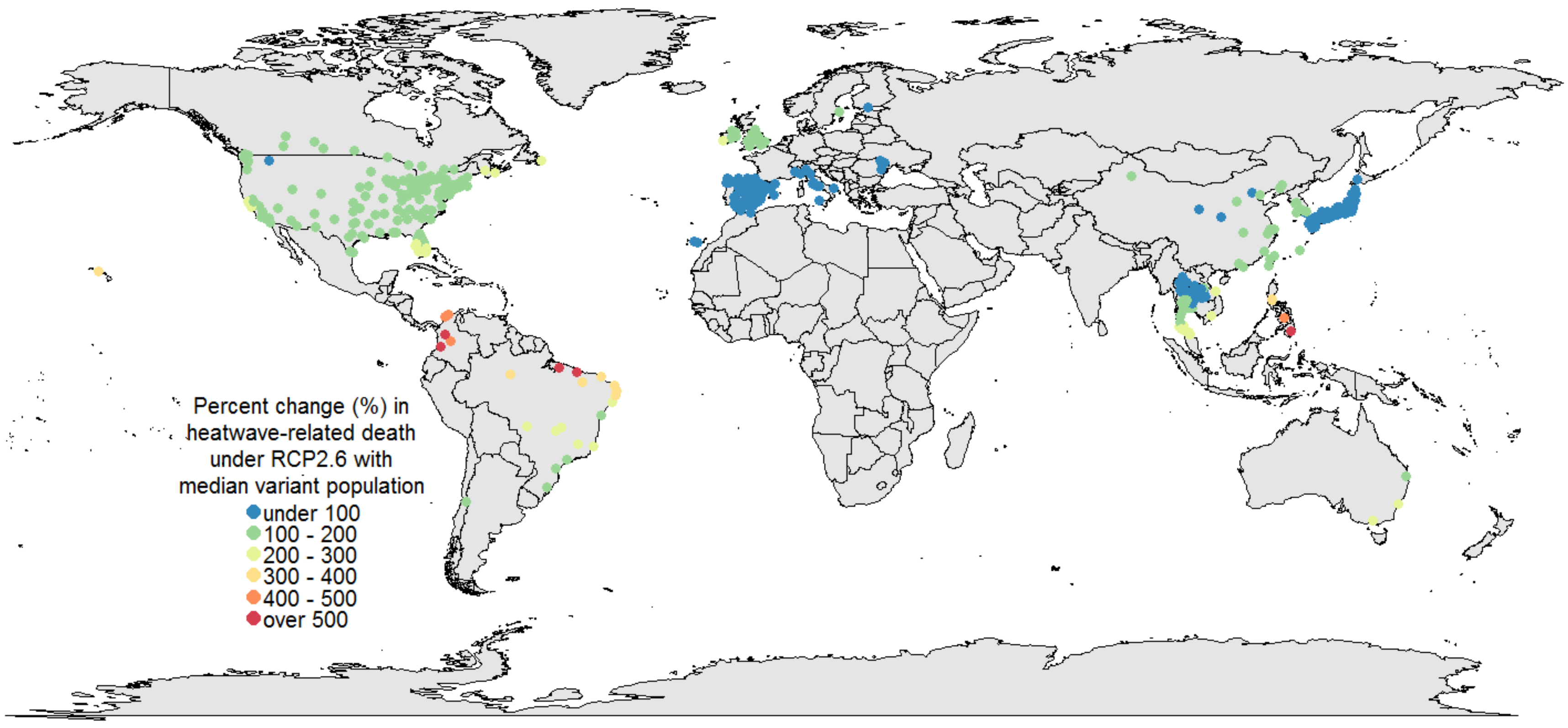

Fig K: Percent change of heatwave-related excess deaths in period 2031-2080 in comparison to period 1971-2020 under RCP2.6 scenario and median variant population scenario, in 412 communities, without any adaptation to future climate change.

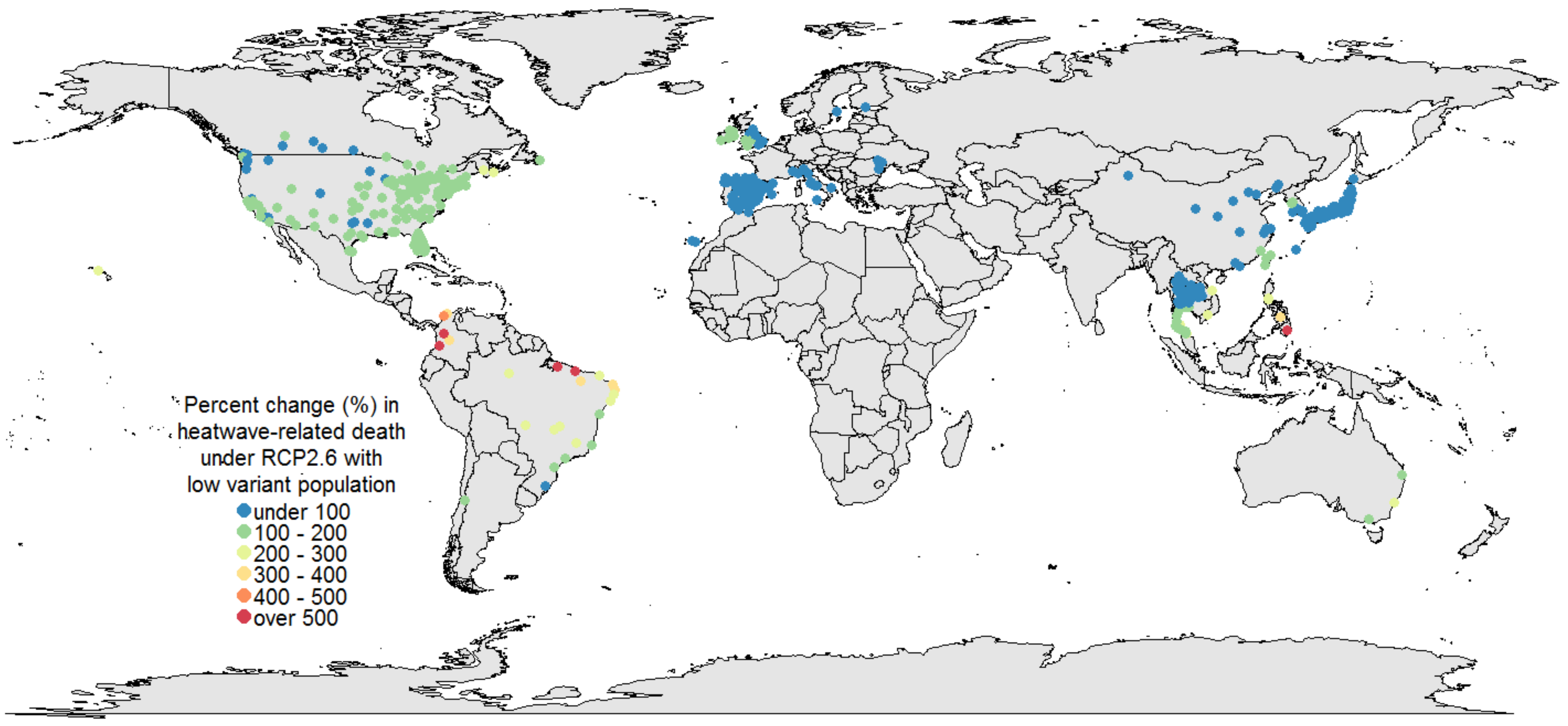

Fig L: Percent change of heatwave-related excess deaths in period 2031-2080 in comparison to period 1971-2020 under RCP2.6 scenario and low variant population scenario, in 412 communities, without any adaptation to future climate change.

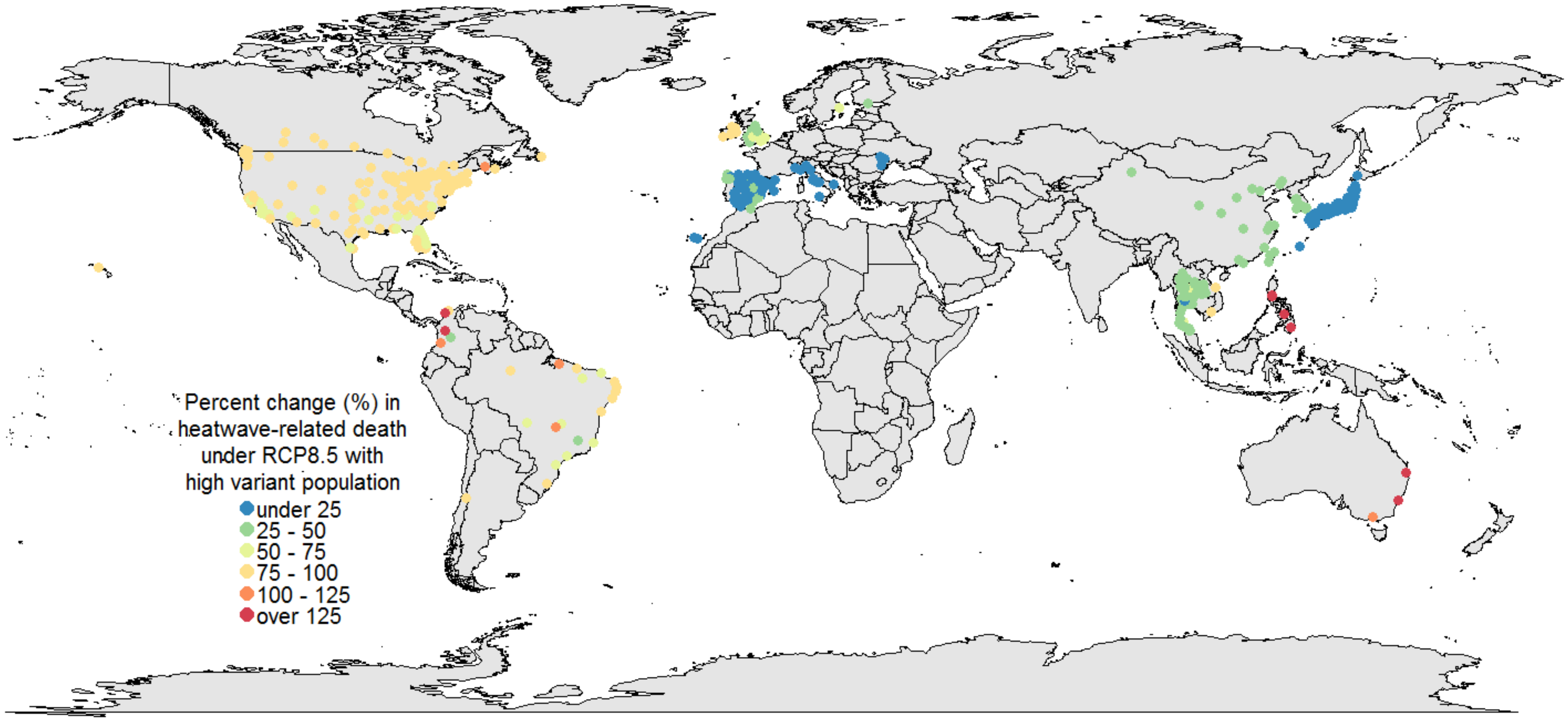

Fig M: Percent change of heatwave-related excess deaths in period 2031-2080 in comparison to period 1971-2020 under RCP8.5 scenario and high variant population scenario, in 412 communities, with full adaptation to future climate change.

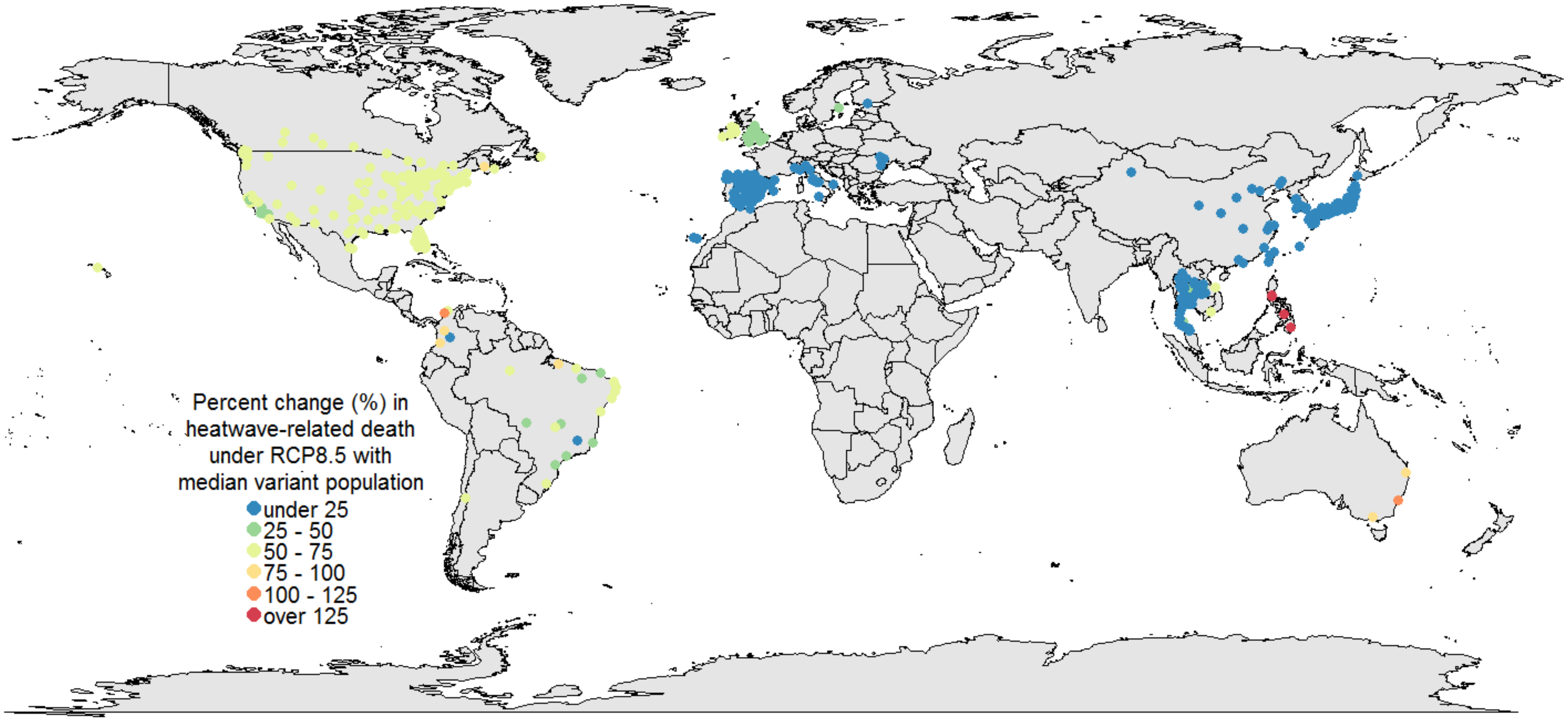

Fig N: Percent change of heatwave-related excess deaths in period 2031-2080 in comparison to period 1971-2020 under RCP8.5 scenario and median variant population scenario, in 412 communities, with full adaptation to future climate change.

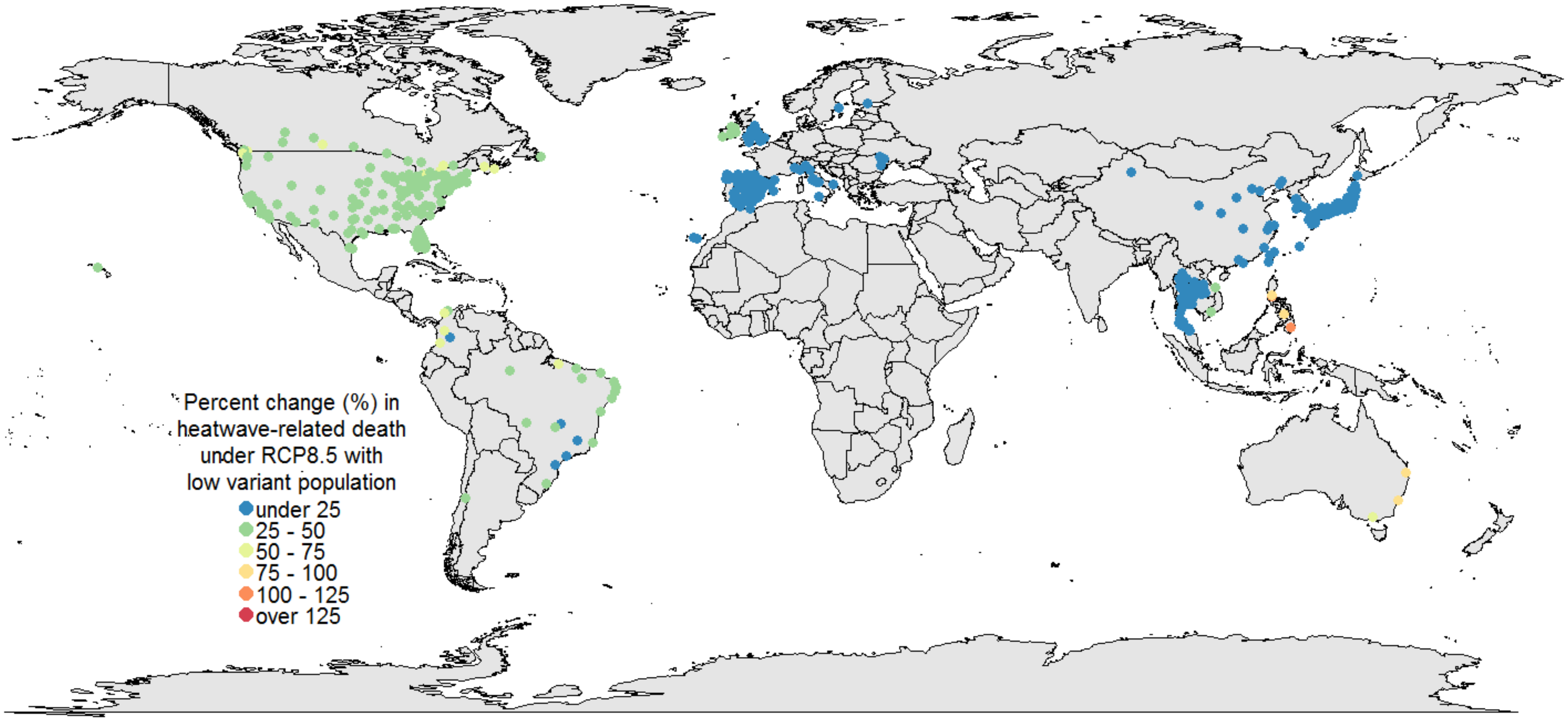

Fig O: Percent change of heatwave-related excess deaths in period 2031-2080 in comparison to period 1971-2020 under RCP8.5 scenario and low variant population scenario, in 412 communities, with full adaptation to future climate change.

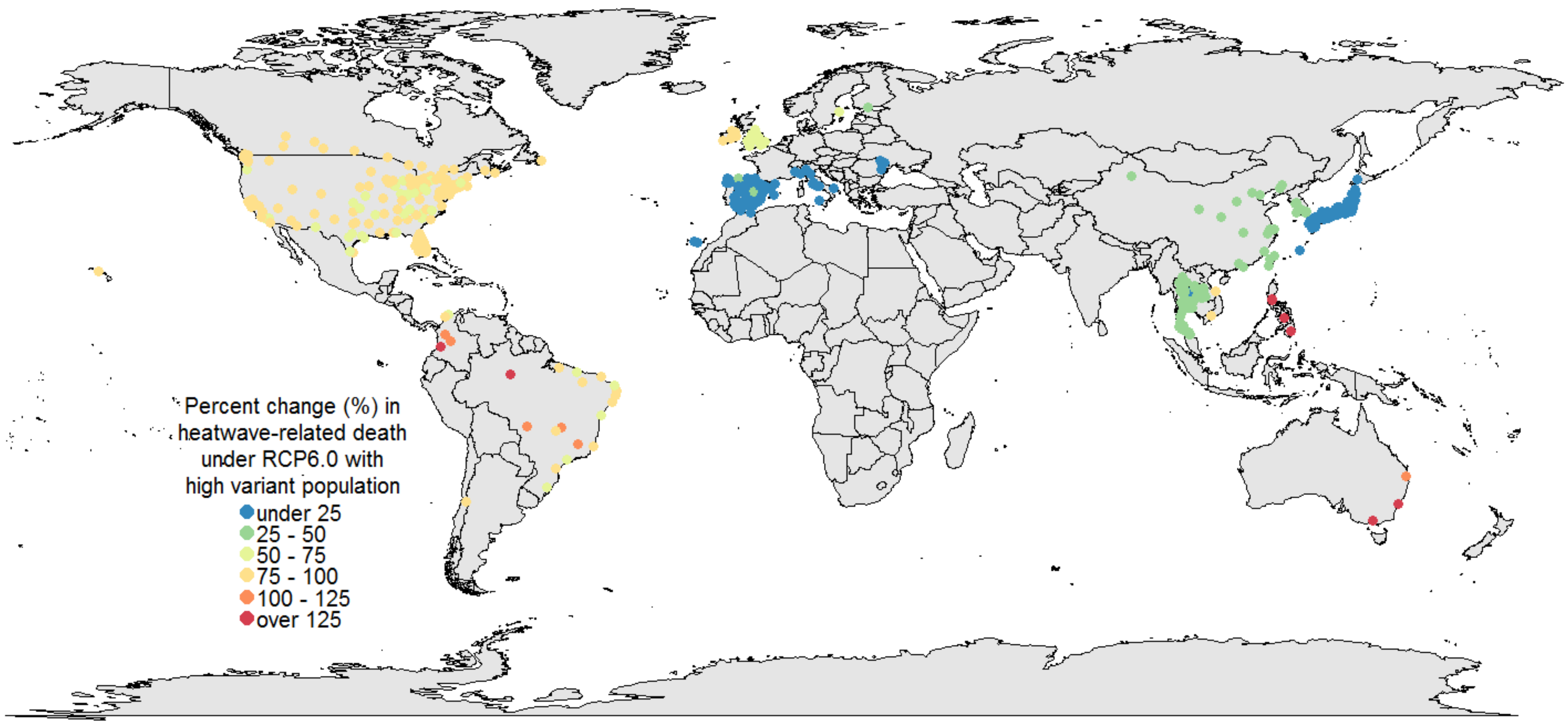

Fig P: Percent change of heatwave-related excess deaths in period 2031-2080 in comparison to period 1971-2020 under RCP6.0 scenario and high variant population scenario, in 412 communities, with full adaptation to future climate change.

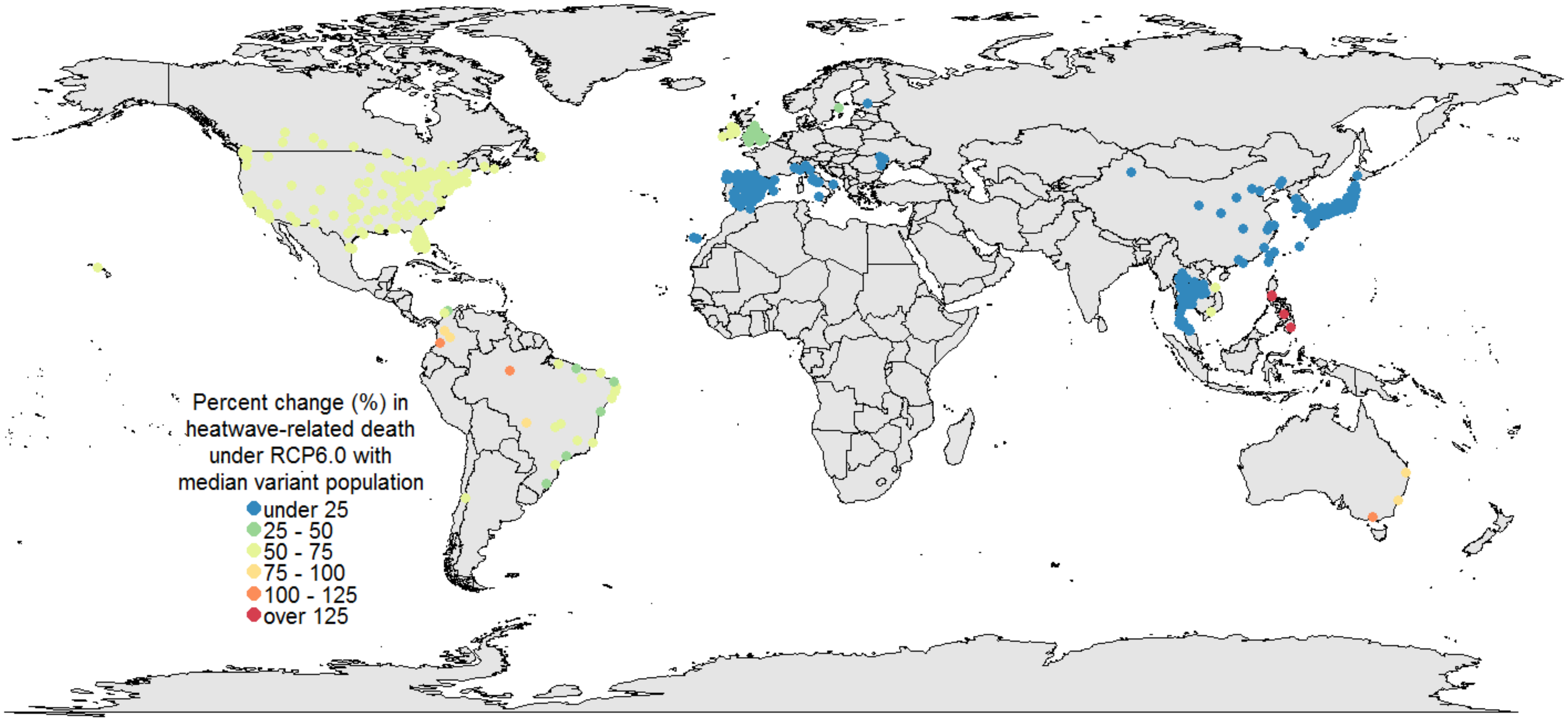

Fig Q: Percent change of heatwave-related excess deaths in period 2031-2080 in comparison to period 1971-2020 under RCP6.0 scenario and median variant population scenario, in 412 communities, with full adaptation to future climate change.

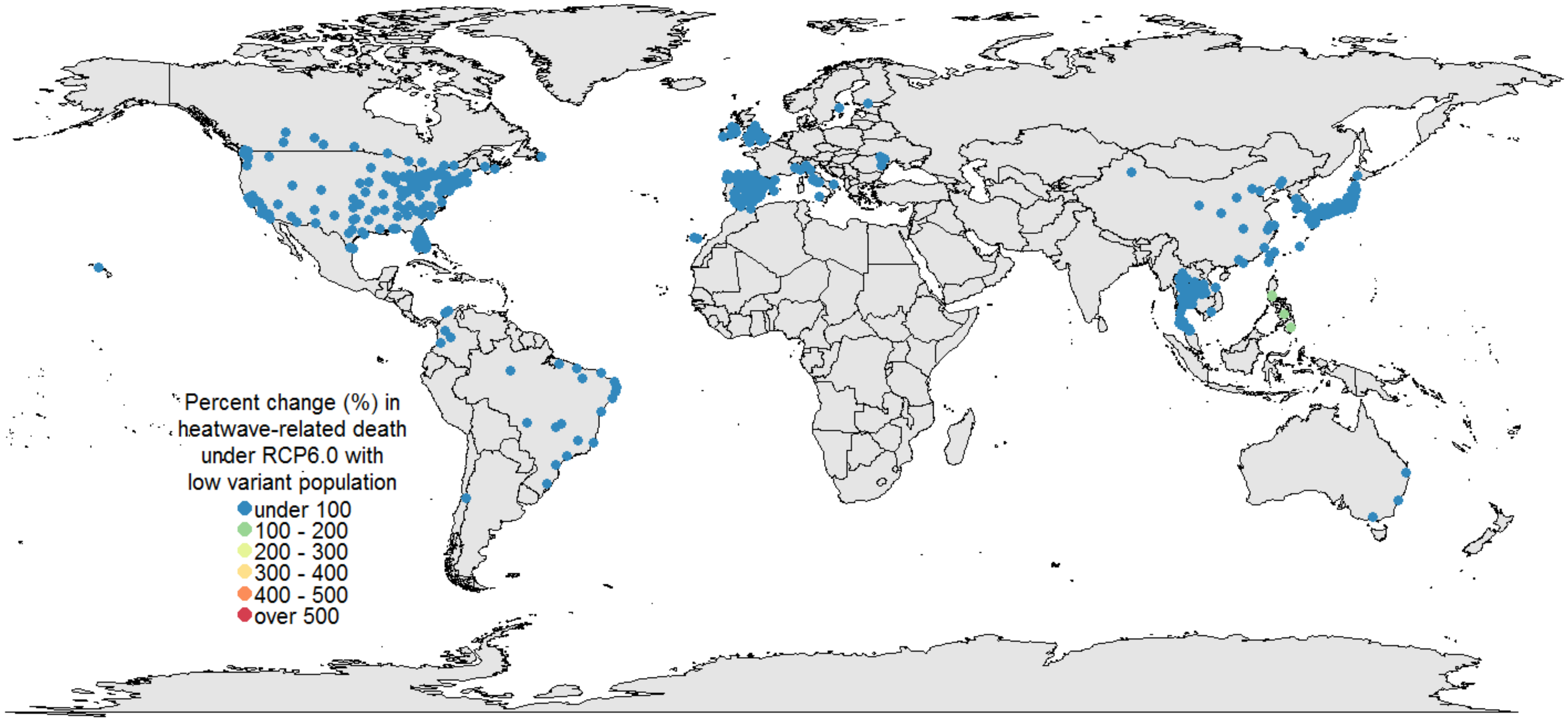

Fig R: Percent change of heatwave-related excess deaths in period 2031-2080 in comparison to period 1971-2020 under RCP6.0 scenario and low variant population scenario, in 412 communities, with full adaptation to future climate change.

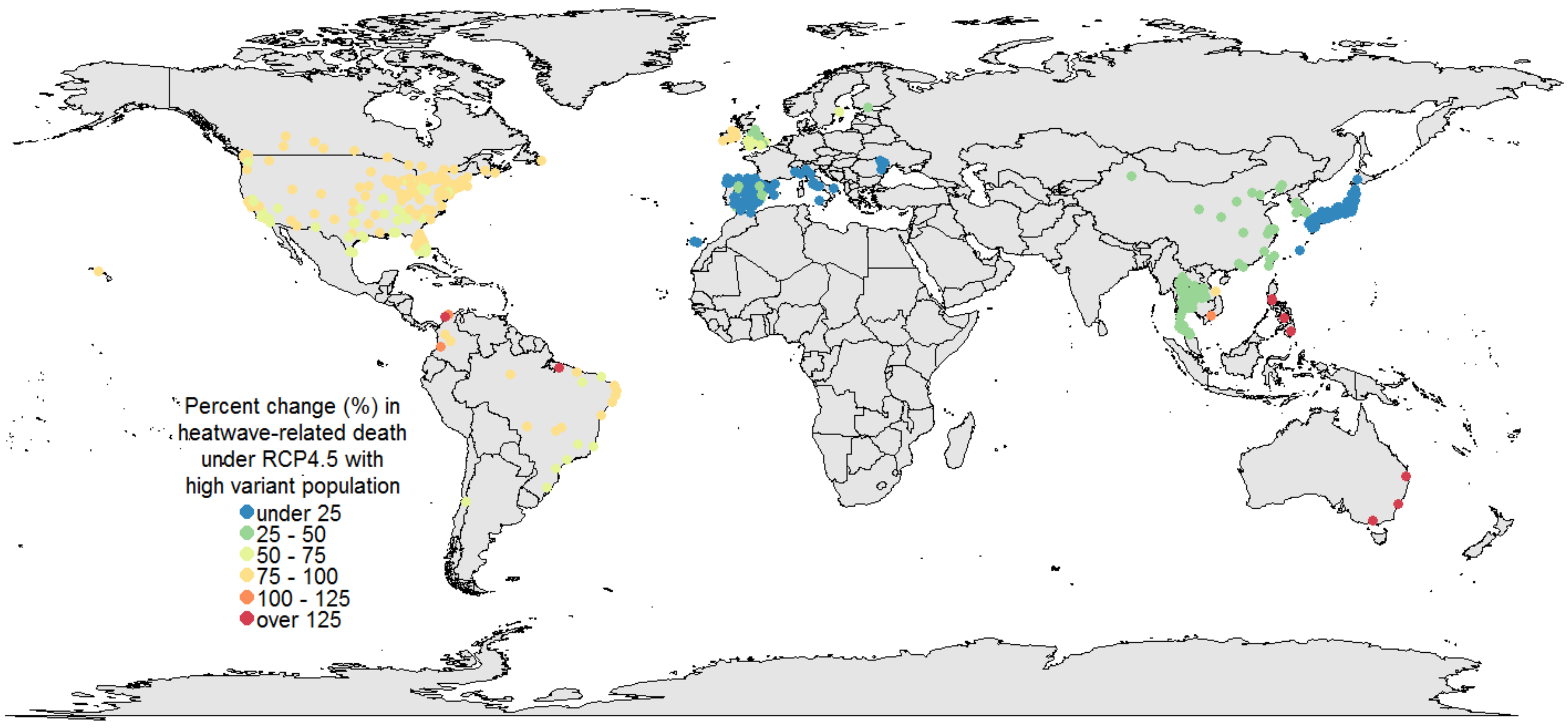

Fig S: Percent change of heatwave-related excess deaths in period 2031-2080 in comparison to period 1971-2020 under RCP4.5 scenario and high variant population scenario, in 412 communities, with full adaptation to future climate change.

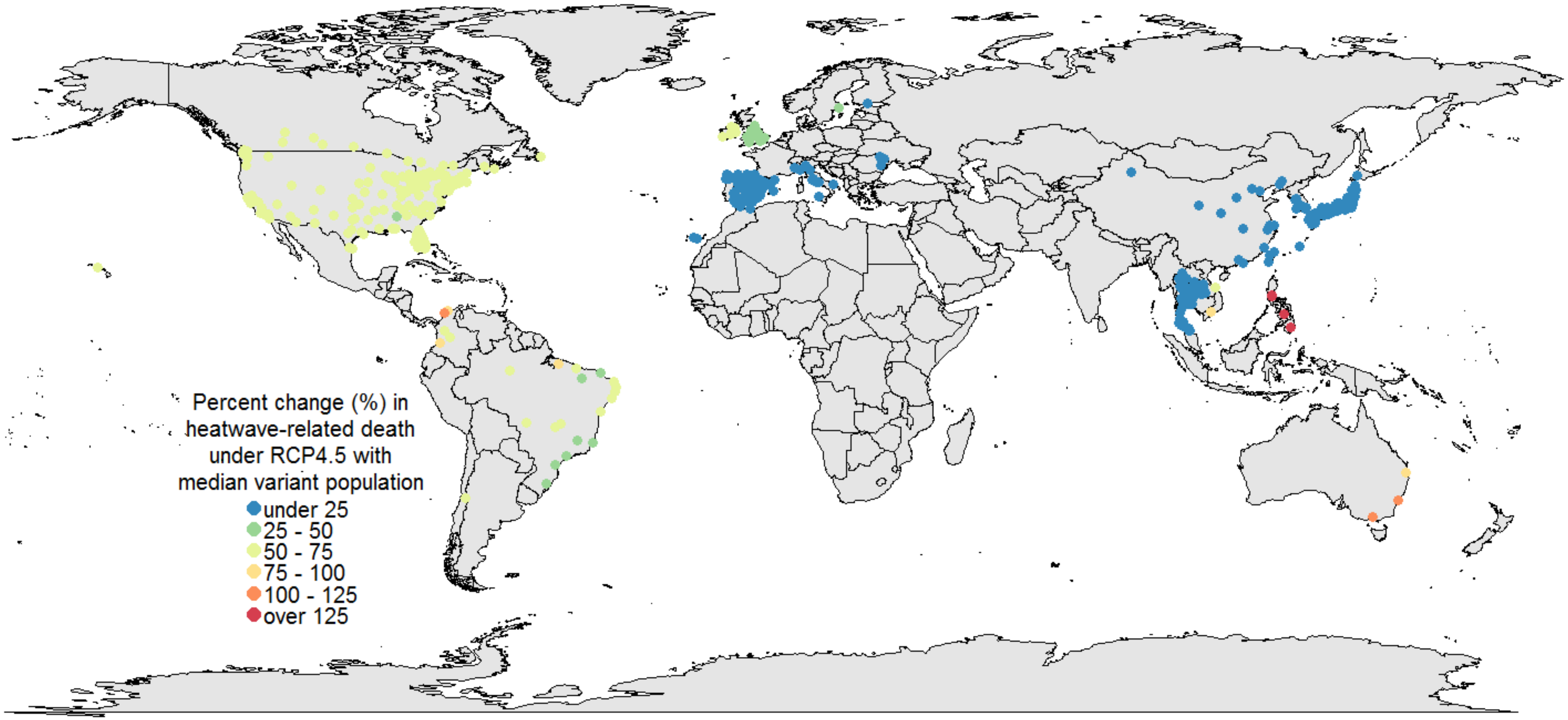

Fig T: Percent change of heatwave-related excess deaths in period 2031-2080 in comparison to period 1971-2020 under RCP4.5 scenario and median variant population scenario, in 412 communities, with full adaptation to future climate change.

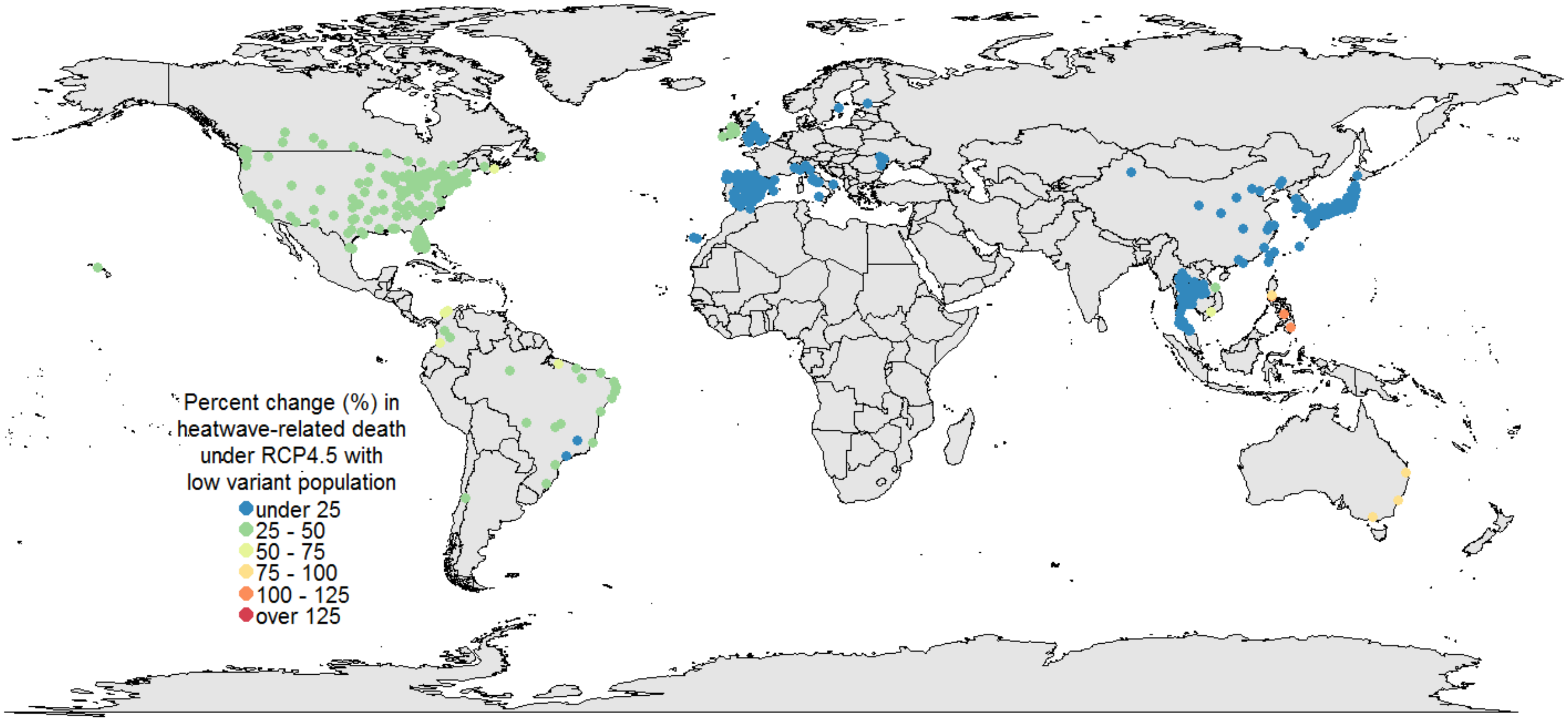

Fig U: Percent change of heatwave-related excess deaths in period 2031-2080 in comparison to period 1971-2020 under RCP4.5 scenario and low variant population scenario, in 412 communities, with full adaptation to future climate change.

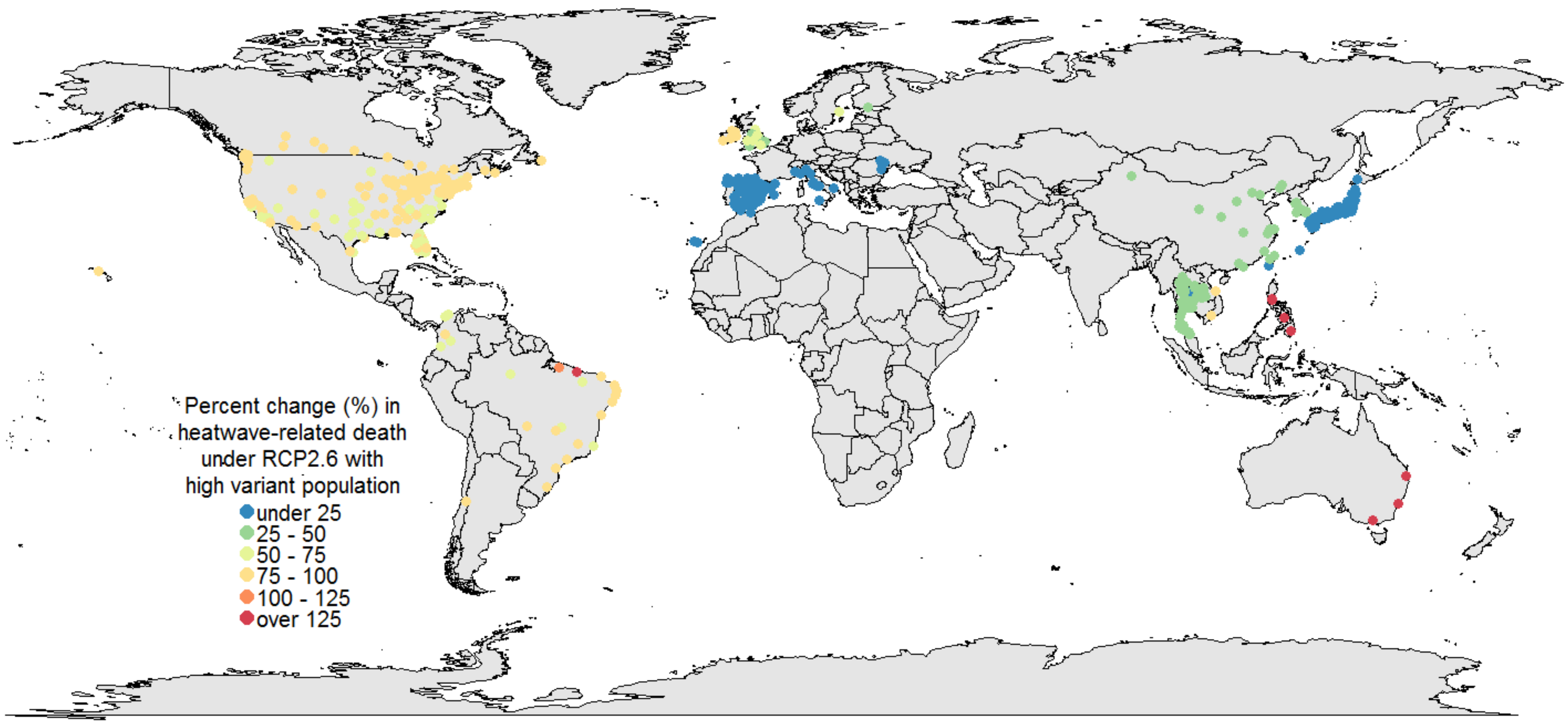

Fig V: Percent change of heatwave-related excess deaths in period 2031-2080 in comparison to period 1971-2020 under RCP2.6 scenario and high variant population scenario, in 412 communities, with full adaptation to future climate change.

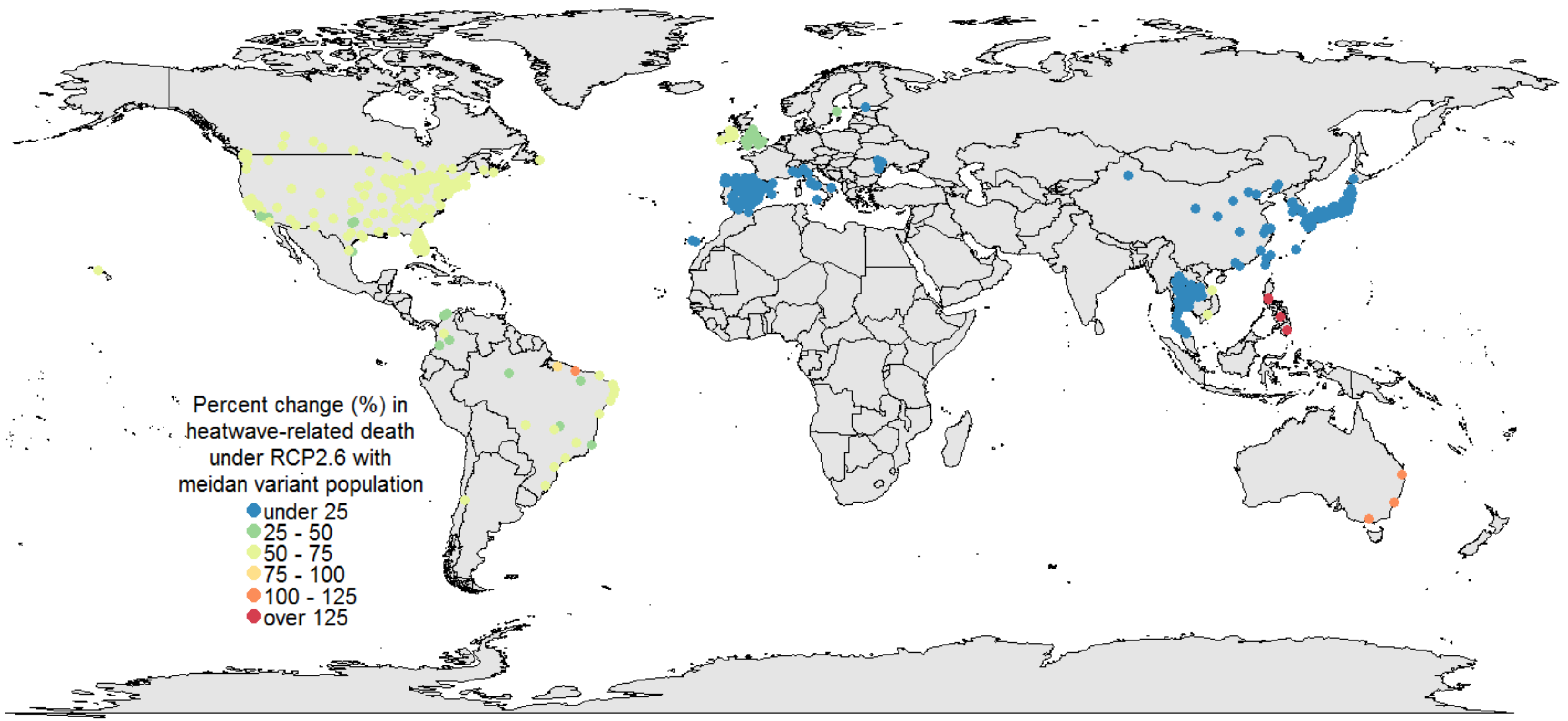

Fig W: Percent change of heatwave-related excess deaths in period 2031-2080 in comparison to period 1971-2020 under RCP2.6 scenario and median variant population scenario, in 412 communities, with full adaptation to future climate change.

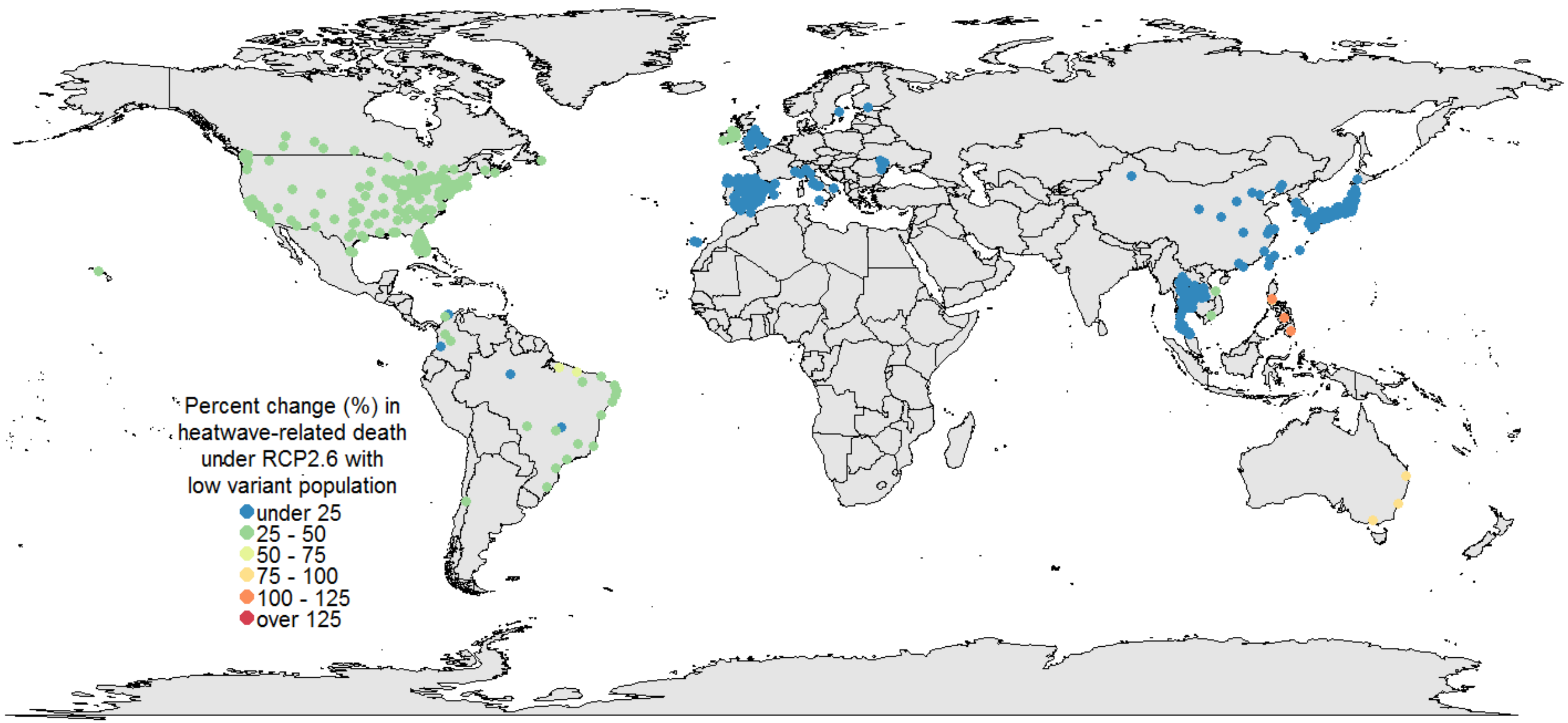

Fig X: Percent change of heatwave-related excess deaths in period 2031-2080 in comparison to period 1971-2020 under RCP2.6 scenario and low variant population scenario, in 412 communities, with full adaptation to future climate change.

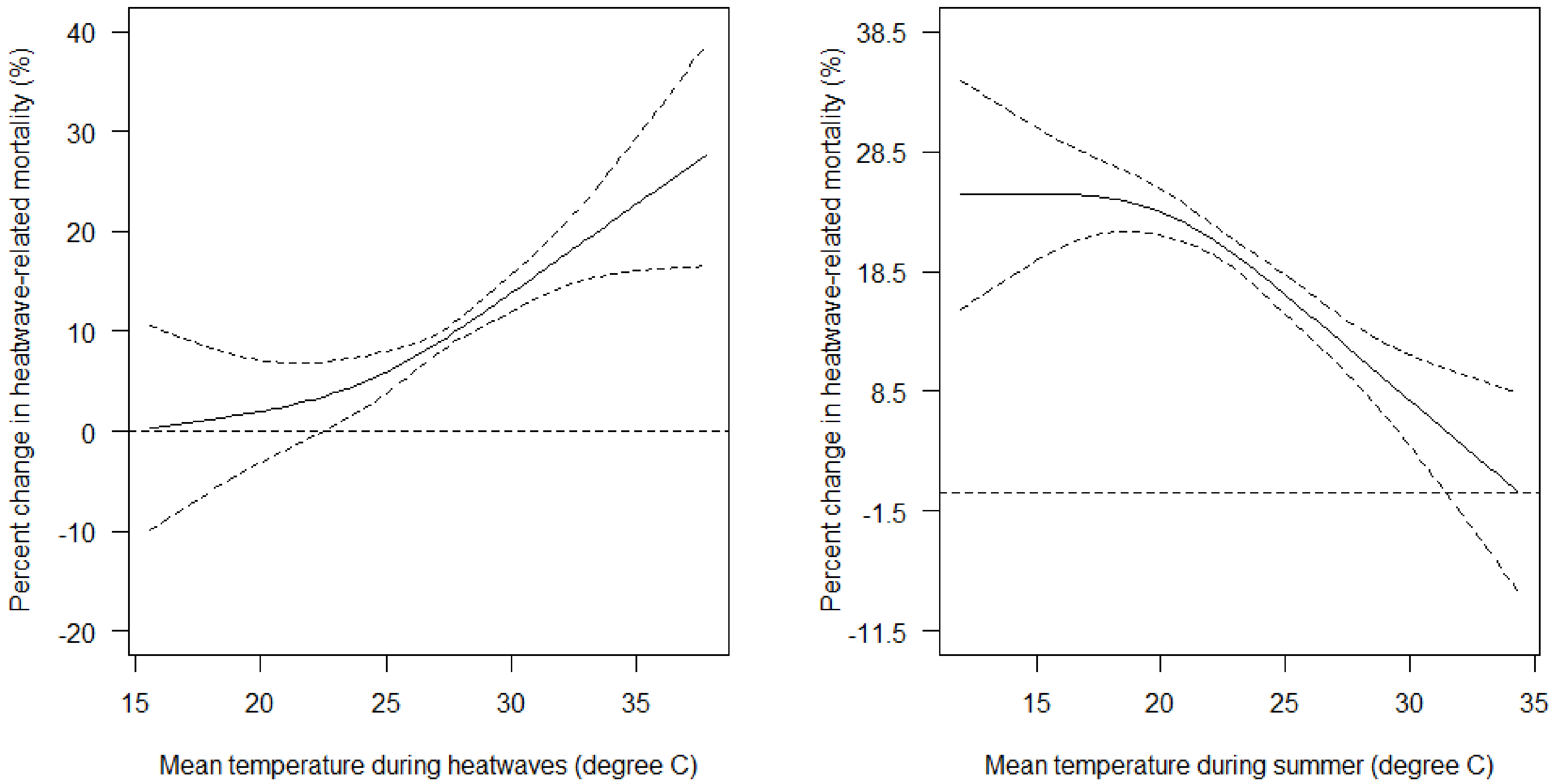

Fig Y: The percent change (and 95%CI) of heatwave-related mortality associated with heatwave intensity (left) and average summer temperature. The dash lines indicate 95%CI.
